# Network pharmacology exploration reveals endothelial inflammation as a common mechanism for stroke and coronary artery disease treatment of Danhong injection

**DOI:** 10.1038/s41598-017-14692-3

**Published:** 2017-11-13

**Authors:** Ming Lyu, Chun-Lin Yan, Hai-Xin Liu, Tai-Yi Wang, Xin-Hui Shi, Jin-Ping Liu, John Orgah, Guan-Wei Fan, Ji-Hong Han, Xiao-Ying Wang, Yan Zhu

**Affiliations:** 1 Tianjin State Key Laboratory of Modern Chinese Medicine, Tianjin University of Traditional Chinese, Tianjin, China; 2 Research and development center of TCM, Tianjin International Joint Academy of Biomedicine, Tianjin, China; 3 Medical Experiment Center, The First Teaching Hospital of Tianjin University of Traditional Chinese Medicine, Tianjin, China; 4 College of Life Sciences, Nankai University, Tianjin, China; 5 Massachusetts General Hospital, Harvard Medical School, Boston, USA

## Abstract

Although Danhong injection (DHI) is the most widely prescribed Chinese medicine for both stroke and coronary artery disease (CAD), its underlying common molecular mechanisms remain unclear. An integrated network pharmacology and experimental verification approach was used to decipher common pharmacological mechanisms of DHI on stroke and CAD treatment. A compound-target-disease & function-pathway network was constructed and analyzed, indicating that 37 ingredients derived from DH (*Salvia miltiorrhiza* Bge., *Flos Carthami tinctorii* and DHI) modulated 68 common targets shared by stroke and CAD. In-depth network analysis results of the top diseases, functions, pathways and upstream regulators implied that a common underlying mechanism linking DHI’s role in stroke and CAD treatment was inflammatory response in the process of atherosclerosis. Experimentally, DHI exerted comprehensive anti-inflammatory effects on LPS, ox-LDL or cholesterol crystal-induced NF-κB, c-jun and p38 activation, as well as IL-1β, TNF-α, and IL-10 secretion in vascular endothelial cells. Ten of 14 predicted ingredients were verified to have significant anti-inflammatory activities on LPS-induced endothelial inflammation. DHI exerts pharmacological efficacies on both stroke and CAD through multi-ingredient, multi-target, multi-function and multi-pathway mode. Anti-endothelial inflammation therapy serves as a common underlying mechanism. This study provides a new understanding of DHI in clinical application on cardiovascular and cerebrovascular diseases.

## Introduction

The 2017 updated Statistics of American Heart Association (AHA) reported that coronary artery disease (CAD) and ischemic stroke ranked as number 1st and 2nd top causes of cardiovascular diseases (CVDs) estimated for the global burden of disease^1^. They not only share a number of malfunctions such as inflammation^2,3^, dysregulated immune system^4^, thrombosis^5^, lipid metabolism^6^, apoptosis^7^ and necrosis^7^, but also several common disease risks, such as atherosclerosis^8,9^, hypertension^10–12^ and diabetes mellitus^2^. A series of researches, combined with certain system biology analyses, indicating an intimate internal connection between stroke and CAD, which are always interplaying^7,13–20^. In addition, ischemic stroke brain may send indirect cell death signals to the heart^7^. Furthermore, Inflammatory response is well recognized as a critical contributor for the development and complications of atherosclerosis cardiovascular disease (ASCVD), including myocardial infarction (MI), heart failure and stroke, which involve complex interactions between multiple biological processes^21,22^. Endothelial cells (ECs) are heterogeneous population that execute many essential physiological processes, which include maintenance of vascular hemostasis and prevention of thrombotic complications by secreting and/or responding to a variety of cytokines and chemokines under pathological conditions^23^. Endothelial inflammation is firmly established as central in the initiation and progression of ASCVD^24–26^. “Endothelial therapy” is proposed as an advanced approach to preserve ECs health, suggesting us to interfere endothelial inflammation at very early time so that to slow down the cardiovascular risk factors^27,28^. In contemporary study, endothelial inflammation therapy has been deemed as a novel therapeutic strategy in ASCVD^29,30^. Treatment with various stimulants activates ECs, causing the production of pro- and anti-inflammatory cytokines and chemokines, which are important in potentiating inflammatory responses cascade, and finally result in increasing cardiovascular event^31^. Lipopolysaccharide (LPS), present in the outer membrane of gram-negative bacteria, plays an important role in triggering the development of endothelial inflammation, which results in tumor necrosis factor (TNF), interleukin-1β (IL-1β), interleukin-10 (IL-10) secretion, nuclear factor-kappa B (NF-κB) nuclear translocation and p38 mitogen activated protein kinase (p38 MAPK) activation^32–34^. Oxidized low-density lipoprotein (ox-LDL) contributes to the atherosclerotic plaque formation and progression by several mechanisms, including the induction of ECs dysfunction and pro-inflammatory cytokines secretion^35,36^. Cholesterol crystal (CHC), a hallmark of atherosclerosis, initiate inflammation via nod-like receptors nucleotide-binding domain and leucine-rich repeat pyrin-3 domain (NLRP3) inflammasome leading to IL-1β and TNF production^37–39^.

Danhong injection (DHI), a Sino Food and Drug Administration (SFDA) approved (Z20026866) Chinese Materia Medica consisting of water-soluble extracts from *Salvia miltiorrhiza* Bge. (Danshen) and *Carthamus tinctorius* L. (Honghua), is prescribed for cardiovascular and cerebrovascular diseases. The formula is originated from traditional Chinese medicine (TCM) theory of “promoting blood circulation and removing blood stasis”. Danshen and Honghua have been used in combination at high frequency in TCM to achieve synergistic therapeutic efficacy for CVD in clinic use in China. From the bench to the bedside, previous studies on DHI for CVDs can mainly documented into three sections: (1) Clinical practices show that DHI has long been extensively used in treatment of stroke and CAD associated diseases (Table 1). (2) Basic researches indicate that DHI can ameliorate cerebral ischemia-reperfusion injury^40–43^, cerebral ischemia damage^44^, myocardial reperfusion injury^45,46^, myocardial hypertrophy^47^, cardiac dysfunction^48^ and cardiac ventricular remodeling^48^. (3) Pharmacological actions of DHI include antioxidation^49,50^, anticoagulation^51^, anti-inflammatory^52–54^, anti-fibrosis^55^, anti-angiogenesis^55^, anti-atherosclerosis^56^ and anti-diabetes^57^ effects. It is also capable of promoting vasodilation^58^, inhibiting vasoconstriction^59^ and hyperlipidemia^60^. Recently, we have reported that the ability of DHI to reinstate arginine vasopressin (AVP) level may be one of its shared mechanisms to protect brain and heart^61^. Although the pleiotropic effects of DHI on both cardio- and cerebral vasculatures are in accordance with the holistic concept of TCM, they also posed a great challenge in identifying individual chemical compounds responsible for the diverse pharmacological mechanisms. Previous studies by us and others have reported certain major active ingredients of DHI, such as phenolic acids, diterpenes and flavonoids, including salvianolic acid B, danshensu, caffeic acid, rosmarinic acid, kaempferol, protocatechuic acid and hydroxysafflor yellow A^51,53,54,58,62–68^.

**Table 1:** Clinical investigations of DHI for CAD and stroke.

Sources	Subject Nos	Diseases	Category	References
Meta-analysis	2660	Acute coronary syndrome	CAD	115
Meta-analysis	7906	Unstable Angina	CAD	116
Meta-analysis	979	Acute myocardial infarction	CAD	117
Systematic reviews	16469	Ischemic stroke	Stroke	118
Clinical research	72	Coronary heart disease unstable angina	CAD	119
Clinical research	54	Coronary heart disease	CAD	52
Clinical research	100	Acute coronary syndrome	CAD	120
Clinical research	246	Acute cerebral infarction	Stroke	94
Clinicaltrials.gov	180	Myocardial Infarction	CAD	—
Clinicaltrials.gov	320	Stroke	Stroke	—
Clinicaltrials.gov	1513	Acute Stroke	Stroke	121
Clinicaltrials.gov	46	Acute Stroke	Stroke	—
Clinicaltrials.gov	160	Unstable Angina Pectoris	CAD	—
Clinicaltrials.gov	870	Chronic Stable Angina	CAD	122

Network pharmacology has been proven to be a dominant paradigm to decipher the complex pharmacological mechanism of action of effective substances of various herbs, herbal pairs, as well as TCM formulae, by incorporating bioinformatics, cheminformatics, and network biology^68–71^. The mystery of herbal pairs and TCM formulae for CVDs is beginning to be revealed with increasing number of studies using network-based approaches, such as compound Danshen formula^72^, compound Saffron formula^73^, Radix Curcumae formula^74^, Shexiang Baoxin pill^75^, QiShenYiQi dropping pill^76^, Huanglian-Jie-Du decoction^77^, ShengMai preparations^78^, Wenxin Keli^79^, Danggui-Honghua pairs^80^ and Danshen-Chuanxiong-Honghua pairs^81^. In addition, an integrated network pharmacology approach is employed to unveil the common and distinct molecular mechanism on several highly correlated diseases, such as CVD-gastrointestinal disorders^82^, psoriasis-rheumatoid arthritis^83^, rheumatoid arthritis-CAD^84^ and stroke-CAD^16,85^, as well as certain TCM subjective theories like Qi-Blood syndrome^86^, Cold-Hot syndrome^87–89^. Combing system-level investigation with experimental validation, these studies facilitate discovering the potential active ingredients and action mechanisms of TCM.

According to the description above, DHI is an ideal TCM in the application of treatment to both stroke and CAD. However, the shared underlying pharmacological mechanisms of DHI on stroke and CAD treatment remain unclear. In this research, we exert a pharmacology network and experimental verification combination method to decipher the potential active ingredients and common key targets, functions, pathways and upstream regulators of DHI in treating stroke and CAD.

## Results

**Figure 1:**
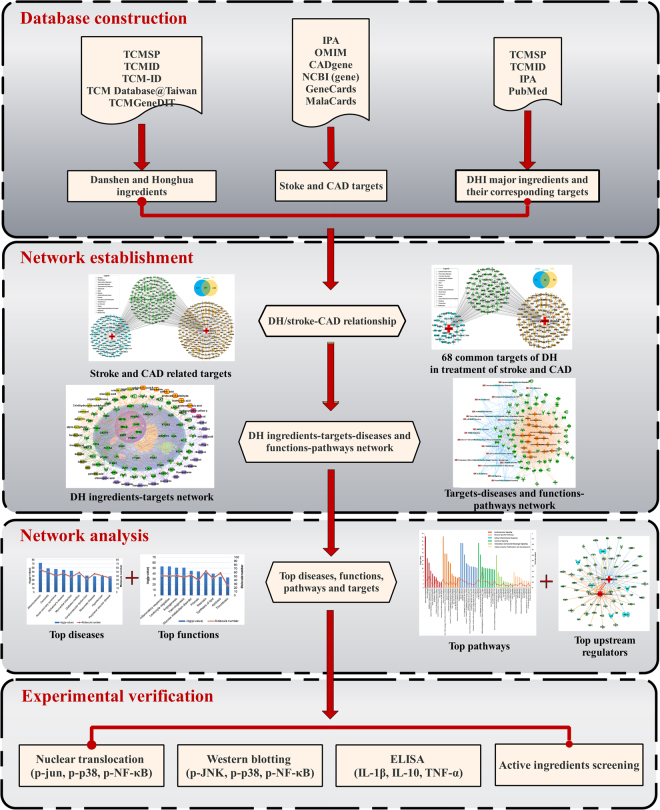
Workflow for DHI co-treatment of both stroke and CAD.

A pharmacology network and experimental verification combination approach was introduced in this study, deciphering the potential curative effects and pharmacology actions of DHI in both stroke and CAD treatment, which involves four steps in a workflow (Fig. 1): (1) the ingredients of prescription along with their corresponding targets and diseases associated targets were identify by various databases; (2) the relationship of compound-target-pathway-disease & function were constructed by interaction networks; (3) the key targets, top functions, top canonical pathways and top upstream regulators were uncovered by network analysis; (4) the efficacy of prescription was validated and the accuracy of network analysis was guaranteed by experimental verification.

### Identification of common targets of stroke and CAD by DH

**Figure 2:**
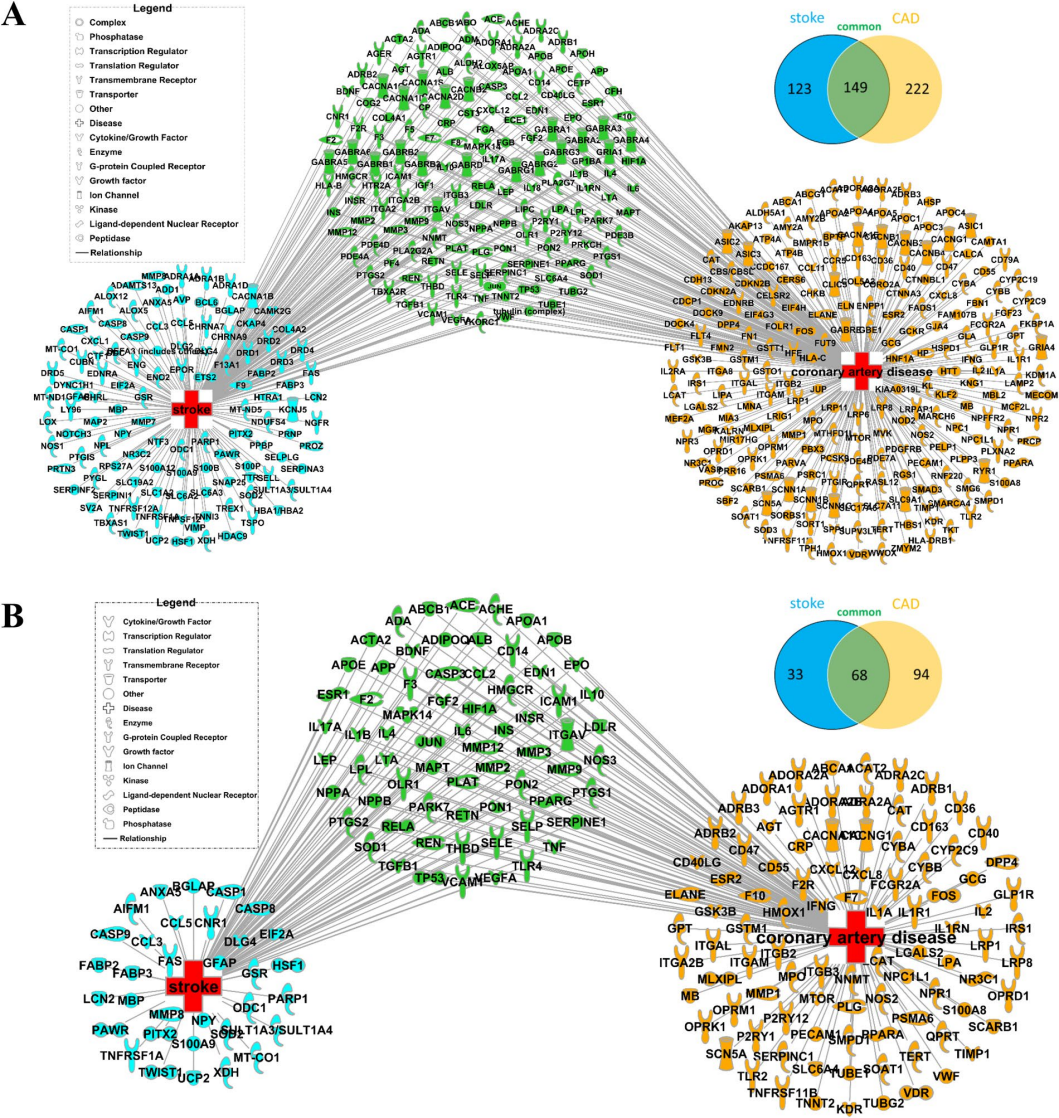
Common targets of DH in the treatment of stroke and CAD. (**A)** Unique and shared disease targets for stroke and CAD. The network depicted 123 unique targets related to stroke (light blue) and 222 unique targets related to CAD (deep yellow) with 149 targets shared by both (green). **(B)** Common targets of DH in treating for stroke and CAD. The network described 33 unique stroke targets (pale blue) and 94 unique CAD targets (deep yellow) with 68 common targets shared by both. Venn diagrams showing the number of shared and unique targets by stroke and CAD were also represented.

A total of 494 targets were identified from 272 stroke-related targets and 371 CAD-related targets by Ingenuity Pathway Analysis (IPA). Among them, 149 were shared by both stroke and CAD, which accounted for 54.8% of the stroke-related targets and 40.2% of the CAD-related targets (Fig. 2A). Among the 494 targets, a total of 195 targets were found to be associated with DH (Danshen, Honghua and DHI) ingredients, with 101 stroke-related and 162 CAD-related targets (Fig. 2B). Sixty-eight of these targets were common for both stroke (67.3%) and CAD (42.0%), which were listed in Supplementary Table S2 and were the focus of our following analysis.

### Establishment and analysis of compound-target-disease & function-pathway network

**Figure 3:**
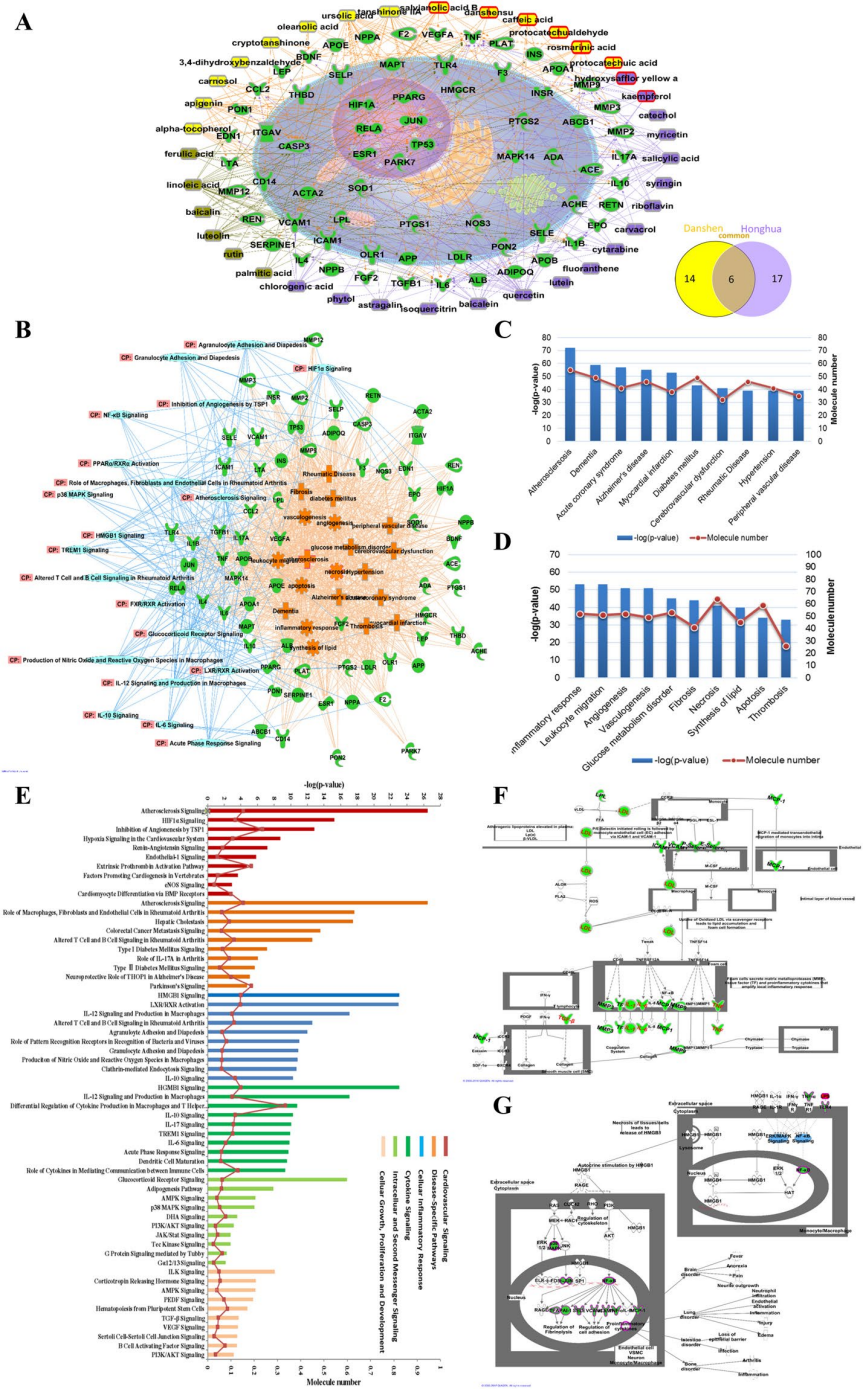
Analysis of compound-target-disease & function-pathway network of DH. (**A)** DH ingredient-target network. Fourteen Danshen ingredients (yellow), 17 Honghua ingredients (violet) and six shared ingredients from these two herbs (gray) were presented, which cooperatively modulate the 68 common intracellular targets. Venn diagram showed the unique and shared numbers of ingredients from DH. Ingredients with red border were identified from DHI. **(B)** Target-disease & function-pathway network. Top 10 diseases, top 10 functions (orange) and top 20 pathways (light blue) correlative with the 68 common (green) targets were shown. **(C** and **D)** Function and disease classification by IPA. The order of top 10 diseases and top 10 functions were ranked from left to right by −log(p-value). **(E)** Six categories with the top 60 expanded pathways list including Cardiovascular signaling, Diseases-specific pathways, Cellular inflammatory response, Cytokine signaling, Intracellular and second message signaling and Cellular growth, proliferation, and development were shown. The order of importance was ranked from top to bottom by −log(p-value). **(F** and **G)** The detailed signaling pathway of atherosclerosis signaling and HMGB1 signaling contain certain highlight targets extracted from the 68 common targets were shown.

For the purpose of interpreting potential pharmacological effects in treatment for both stroke and CAD by DH, compound-target combined with target-disease & function-pathway networks were constructed. DH ingredient-multiple target network was established, elucidating the 37 DH ingredients (20 were derived from Danshen, 23 were derived from Honghua, and six were from both herbs) modulated the 68 common targets. Details of the 37 DH ingredients were shown (see Supplementary Table S3). We integrated the compound-target network into a model cell to demonstrate the possible cellular locations and multi-target biological processes of DH ingredients. The interactions were multidimensional in nature, in which both “one ingredient-multiple targets” and “one target-multiple ingredients” phenomena were revealed (Fig. 3A). In combination with diverse modules of IPA, an integrated target-disease & function-pathway network containing the most relevant pathways, most correlative diseases, and most related functions were built to clarify the biological process and molecular mechanisms of DH acting on the 68 common targets (Fig. 3B).

The diseases, functions and pathways were ranked respectively to discover and distinguish the significance by using a p-value score according to the Fisher’s exact test algorithm (Fig. 3C–E). The most impacted diseases by DH, in an order of descending −log(p-value) score, were atherosclerosis, dementia, acute coronary syndrome, Alzheimer’s disease, myocardial infarction, diabetes mellitus, cerebrovascular dysfunction, rheumatic disease, hypertension and peripheral vascular disease. Among them, the atherosclerosis ranked the highest with a −log(p-value) score of 72. DH was predicted to influence multiple functions including, in an order of descending −log(p-value) score: inflammatory response, leukocyte migration, angiogenesis, vasculogenesis, glucose metabolism disorder, fibrosis, necrosis, synthesis of lipid, apoptosis and thrombosis. Among them, the inflammatory response ranked the highest with a −log(p-value) score of 52. Based on the analyses of the 68 common targets, top diseases and top functions obtained above, we sorted and ranked pathways into six categories, including cardiovascular signaling, diseases-specific pathways, cellular inflammatory response, cytokine signaling, intracellular and second message signaling and Cellular growth/proliferation/development. The expanded correlative top 60 pathways were obtained (Fig. 3B). According to IPA canonical pathways analysis, atherosclerosis signaling was considered as the most essential pathway in cardiovascular signaling while HMGB1 signaling was the most critical players in cellar inflammatory response (Fig. 3E). The detailed pathways of the atherosclerosis signaling and HMGB1 signaling including the identified molecular targets were generated by IPA (Fig. 3G,F).

**Figure 4:**
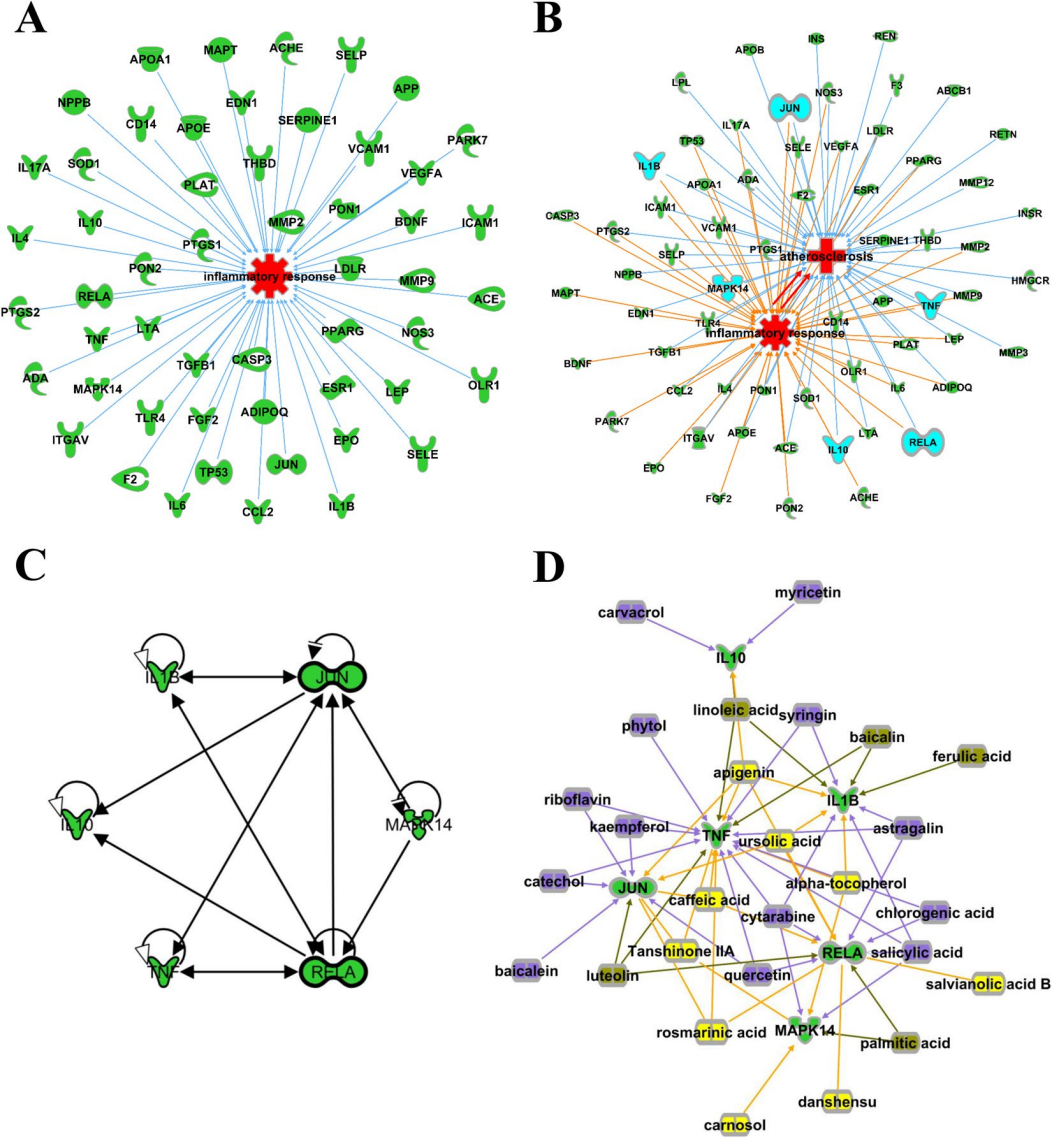
Upstream regulators of DH action shared by inflammatory response and atherosclerosis. **(A)** Fifty targets related to inflammatory response extracted from the 68 common targets. **(B)** The relationship including six key upstream regulators between inflammatory response and atherosclerosis. **(C)** The PPI network between the six key upstream regulators. **(D)** The synergetic effects of 27 DH ingredients on the six key upstream regulators. This sub-network was drawn from Fig. 3A, which contains 9 Danshen (yellow), 13 Honghua (violet) and 5 shared ingredients from both herbs (gray).

To demonstrate the key role of inflammation in DH treatment for both stroke and CAD, we extracted the targets that were related to inflammatory response from the 68 common targets. Fifty of the 68 were positive by IPA (Fig. 4A). The close relationship involved in multiple shared and unique targets between inflammatory response and atherosclerosis was also confirmed in the crosstalk network (Fig. 4B **)**. Next, the top 20 upstream regulators and their corresponding targets were obtained from the 50 targets in correlation with inflammatory response based on the IPA “Core analysis” platform (Table 2). Among them, three inflammatory cytokines, IL1B (IL-1β), TNF (TNF-α) and IL10 (IL-10), and three transcription regulators, RELA (NF-κB), JUN (c-jun) and MAPK 14 (p38 MAPK) were shown by IPA to have a cross-talking protein-protein interaction (PPI) and were selected for further experimental validation (Fig. 4C **)**. These six inflammatory molecules were modulated by 27 DH ingredients in a cooperative manner (Fig. 4D) and highlighted in inflammatory response and atherosclerosis cross-talk network (Fig. 4B).

**Table 2:** Top 20 upstream regulators and their downstream regulated targets.

No.	Upstream Regulator	Molecule Type	p-value of overlap	Target molecules in dataset	Mechanistic Network
**1**	APOE	transporter	2.36E-43	ADIPOQ,APOA1,APOE,APP,CASP3,EDN1,ICAM1,IL10,IL17A,IL1B,IL4,IL6,JUN,LDL,LDLR,LEP,MAPT,MMP2,MMP9,NOS3,PPARG,PTGS2,RELA, SELE,SERPINE1,SOD1,TGFB1,TNF,VCAM1	47 (21)
**2**	IL1B	cytokine	1.86E-42	ACHE,APOE,APP,CASP3,CCL2,CD14,EDN1,EPO,ESR1,FGF2,ICAM1,IL10,IL17A,IL1B,IL6,ITGAV,JUN,LDLR,LEP,LTA,MAPK14,MAPT,MMP2, MMP9,NOS3,OLR1,PLAT,PPARG,PTGS1,PTGS2,RELA,SELE,SERPINE1, TGFB1,THBD,TLR4,TNF,VCAM1,VEGFA	50 (22)
**3**	TNF	cytokine	4.9E-41	ACE,ADIPOQ,APOA1,APOE,APP,BDNF,CASP3,CCL2,CD14,EDN1,EPO, ESR1,FGF2,ICAM1,IL10,IL17A,IL1B,IL4,IL6,ITGAV,JUN,LDLR,LEP, MAPK14,MMP2,MMP9,NOS3,NPPB,OLR1,PLAT,PPARG,PTGS1,PTGS2, RELA,SELE,SELP,SERPINE1,SOD1,TGFB1,THBD,TLR4,TNF,TP53, VCAM1,VEGFA	49 (16)
**4**	LEP	growth factor	1.26E-38	ADIPOQ,APOA1,APP,BDNF,CASP3,CCL2,CD14,EDN1,ESR1,ICAM1,IL10,IL1B,IL4,IL6,JUN,LDLR,LEP,MMP2,NOS3,PLAT,PPARG,PTGS2,SELE, SELP,SERPINE1,SOD1,TGFB1,TNF,TP53,VCAM1,VEGFA	48 (17)
**5**	PPARG	ligand-dependent nuclear receptor	4.67E-36	ADIPOQ,APOA1,APOE,APP,CCL2,EDN1,ICAM1,IL10,IL17A,IL1B,IL4,IL6,JUN,LDLR,LEP,MMP9,NOS3,NPPB,OLR1,PPARG,PTGS2,RELA,SELE, SERPINE1,SOD1,TLR4,TNF,TP53,VCAM1,VEGFA	47 (23)
**6**	TGFB1	growth factor	1.26E-34	ACE,ADIPOQ,APOE,APP,BDNF,CASP3,CCL2,CD14,EDN1,FGF2,ICAM1, IL10,IL17A,IL1B,IL4,IL6,ITGAV,JUN,LDLR,LEP,LTA,MAPK14,MMP2,MMP9,NOS3,NPPB,OLR1,PLAT,PPARG,PTGS1,PTGS2,SELE,SELP, SERPINE1,TGFB1,THBD,TLR4,TNF,TP53,VCAM1,VEGFA	46 (18)
**7**	IL6	cytokine	1.25E-33	APOA1,APOE,APP,BDNF,CASP3,CCL2,CD14,EPO,FGF2,ICAM1,IL10, IL17A,IL4,IL6,ITGAV,JUN,LDLR,LEP,MMP2,MMP9,NOS3,PLAT,PON1, PPARG,PTGS2,SERPINE1,TGFB1,TLR4,TNF,TP53,VCAM1,VEGFA	45 (20)
**8**	RELA	transcription regulator	8.51E-33	APOE,APP,CASP3,CCL2,CD14,EDN1,FGF2,ICAM1,IL10,IL1B,IL4,IL6,JUN,LTA,MMP9,NPPB,OLR1,PPARG,PTGS2,RELA,SELE,SELP,TGFB1,TNF, TP53,VCAM1,VEGFA	45 (15)
**9**	IL17A	cytokine	1.72E-32	CCL2,CD14,FGF2,ICAM1,IL10,IL17A,IL1B,IL4,IL6,JUN,LEP,MMP2,MMP9,NOS3,PPARG,PTGS2,SELE,SELP,THBD,TLR4,TNF,VCAM1,VEGFA	41 (22)
**10**	EGR1	transcription regulator	5.49E-32	ACE,ACHE,APOA1,CASP3,CCL2,FGF2,ICAM1,IL1B,IL4,JUN,LDLR,MMP9,PPARG,PTGS2,SERPINE1,SOD1,TGFB1,TLR4,TNF,TP53,VCAM1,VEGFA	50 (19)
**11**	VEGFA	growth factor	9.61E-32	ACE,ACHE,CASP3,CCL2,EDN1,FGF2,ICAM1,IL1B,IL6,ITGAV,MMP2, MMP9,NOS3,PLAT,PTGS1,PTGS2,SELE,SERPINE1,TGFB1,THBD,TNF, TP53,VCAM1,VEGFA	48 (18)
**12**	TLR4	transmembrane receptor	3.87E-27	APP,CCL2,CD14,EDN1,ICAM1,IL10,IL17A,IL1B,IL4,IL6,LTA,MMP9,NOS3,PLAT,PPARG,PTGS2,RELA,SELE,SELP,TGFB1,TLR4,TNF,VCAM1	45 (23)
**13**	FGF2	growth factor	2.73E-25	ACE,BDNF,CCL2,FGF2,ICAM1,IL1B,IL6,JUN,MAPT,MMP2,MMP9,NOS3, PLAT,PPARG,PTGS2,SELE,SERPINE1,TGFB1,TNF,TP53,VCAM1,VEGFA	48 (17)
**14**	F2	peptidase	5.24E-25	CASP3,CCL2,EDN1,EPO,FGF2,ICAM1,IL1B,IL6,JUN,MMP9,NOS3,PLAT, PTGS2,SELE,SELP,SERPINE1,THBD,TNF,VCAM1,VEGFA	48 (19)
**15**	TP53	transcription regulator	3.65E-24	ACE,ADA,APOA1,APOE,APP,CASP3,CCL2,EDN1,ESR1,FGF2,ICAM1,IL10,IL1B,IL4,IL6,JUN,MMP2,MMP9,NOS3,PARK7,PPARG,PTGS1,PTGS2, RELA,SELP,SERPINE1,SOD1,TGFB1,THBD,TNF,TP53,VEGFA	49 (25)
**16**	JUN	transcription regulator	1.62E-23	APOE,APP,BDNF,CCL2,CD14,EDN1,FGF2,ICAM1,IL10,IL1B,IL6,ITGAV, JUN,MMP2,MMP9,PTGS2,SERPINE1,TGFB1,TNF,TP53,VCAM1,VEGFA	47 (19)
**17**	MAPK14	Kinase	8.07E-23	CCL2,EPO,ICAM1,IL10,IL1B,IL4,IL6,JUN,LDLR,MAPK14,MMP9,PTGS2, SOD1,TGFB1,TNF,TP53,VEGFA	49 (21)
**18**	IL10	cytokine	3.6E-22	CASP3,CCL2,CD14,ICAM1,IL10,IL17A,IL1B,IL4,IL6,JUN,MMP2,MMP9, NOS3,PTGS2,SELE,TGFB1,TLR4,TNF,VCAM1,VEGFA	44 (21)
**19**	LDLR	transporter	7.47E-22	APOE,ICAM1,IL10,IL1B,IL6,LDL,LDLR,MMP2,MMP9,NOS3,PPARG,SELE,TNF,VCAM1	47 (20)
**20**	PTGS2	enzyme	1.32E-21	CCL2,ICAM1,IL10,IL1B,IL6,ITGAV,LEP,MMP2,MMP9,NOS3,PPARG, PTGS1,PTGS2,TNF,TP53,VEGFA	48 (22)

All of the results we obtained above imply that the cross-talk between inflammatory response and atherosclerosis may serve as both stroke- and CAD-related common mechanisms for DHI. Since endothelial inflammation is pivotal to the pathobiology of ASCVD, we choose a vascular endothelial cell as a model to experimentally verify the role of DHI in the treatment of both stroke and CAD by targeting the inflammation-atherosclerosis cross-talk.

### Effects of DHI on different stimulant-induced nuclear translocation of c-Jun, p38 MAPK, and NF-κB p65

The dose-range of DHI for *in vitro* cellular study was first determined by CCK-8 assay. DHI at lower than 1/400 dilutions had no significant cytotoxicity in EA.hy926 cells after 24 h treatment (see Supplementary Fig. S1). However, cell viability was affected at above 1:200 dilutions. Therefore, the doses of DHI were chosen between 1/800-1/3200 dilutions in the subsequent experiments.

**Figure 5:**
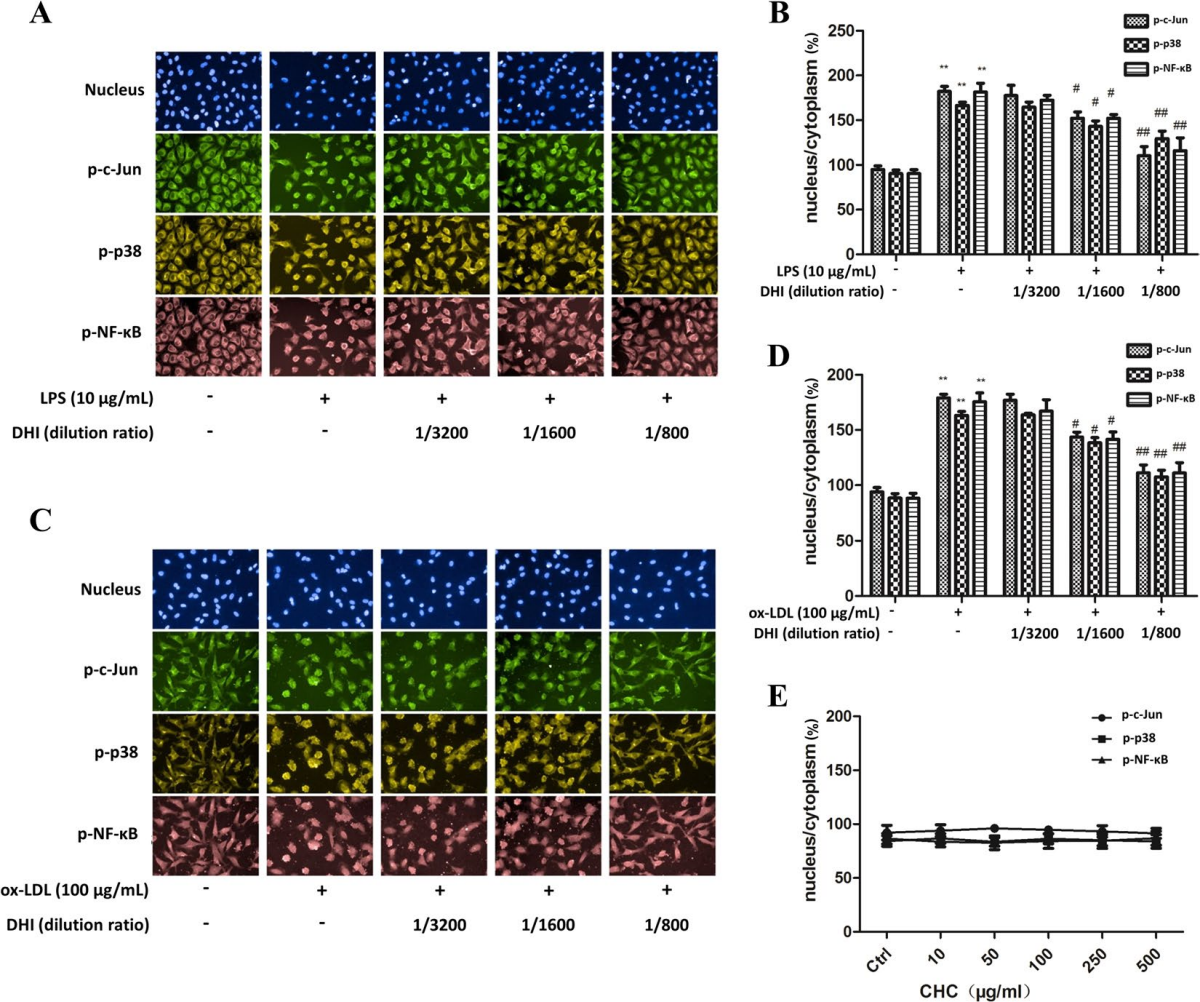
The effects of DHI on nuclear translocation of c-Jun, p38, NF-κB p65 induced by different stimulants. EA.hy926 cells were pre-incubated with DHI for 1 h before adding 10 μg/mL LPS or 100 μg/mL ox-LDL, and cultured for 30 min. **(A)** Representative photo-images and **(B)** summary bar graph of DHI at different dilution ratio (1/3200, 1/1600 and 1/800) on 10 μg/mL LPS induced p-c-Jun, p-p38 or p-NF-κB p65 nuclear translocation. **(C)** Representative photo-images and **(D)** summary bar graph of different dilution ratio of DHI on 100 μg/mL ox-LDL induced p-c-Jun, p-p38, or p-NF-κB p65 nuclear translocation. **(E)** CHC (10–500 μg/mL) on p-c-Jun, p-p38 or p-NF-κB p65 nuclear translocation. Nucleus were stained by Hoechst (blue) and the transcriptional factors were stained by immunolabeled antibodies for p-c-Jun (green), p-p38 (yellow), or p-NF-κB p65 (red). Cells were imaged with the HCA reader using a 20× objective lens with each column reflecting images collected from the respective fluorescent channels using the same optical field. Data are presented as mean ± SD (n = 3). **P < 0.01 versus control; ^#^P < 0.05 versus LPS or ox-LDL group; ^##^P < 0.01 versus LPS or ox-LDL group.

To compare the inflammatory responses in vascular endothelial cells, we stimulated cells with three different stimuluses, LPS, ox-LDL and CHC, and then examined translocation of inflammation-specific transcription factors (TFs), p-c-Jun, p-p38 and p-NF-κB p65 to the nucleus. Dose-response curves of LPS and ox-LDL for stimulation of nuclear translocation of p-c-Jun, p-p38 and p-NF-κB p65 in EA.hy926 cells were shown in Supplementary Fig. S2 whereas CHC at up to 500 μg/ml had no effect (Fig. 5E). Therefore, we further determined the effect of DHI on the activation (translocation) of the inflammatory TFs. As shown by the fluorescence microscopic images from high-content analyzer (HCA) (Fig. 5A,C) and their quantitation (Fig. 5A–D **)**. DHI at dilutions of 1/1600 and 1/800 dose-dependently inhibited LPS (10 μg/mL) and ox-LDL (100 μg/mL)-stimulated nuclear translocation of p-c-Jun, p-p38 and p-NF-κB p65, respectively.

### Effects of DHI on different stimulant-induced phosphorylation of JNK, p38 MAPK, and NF-κB p65

**Figure 6:**
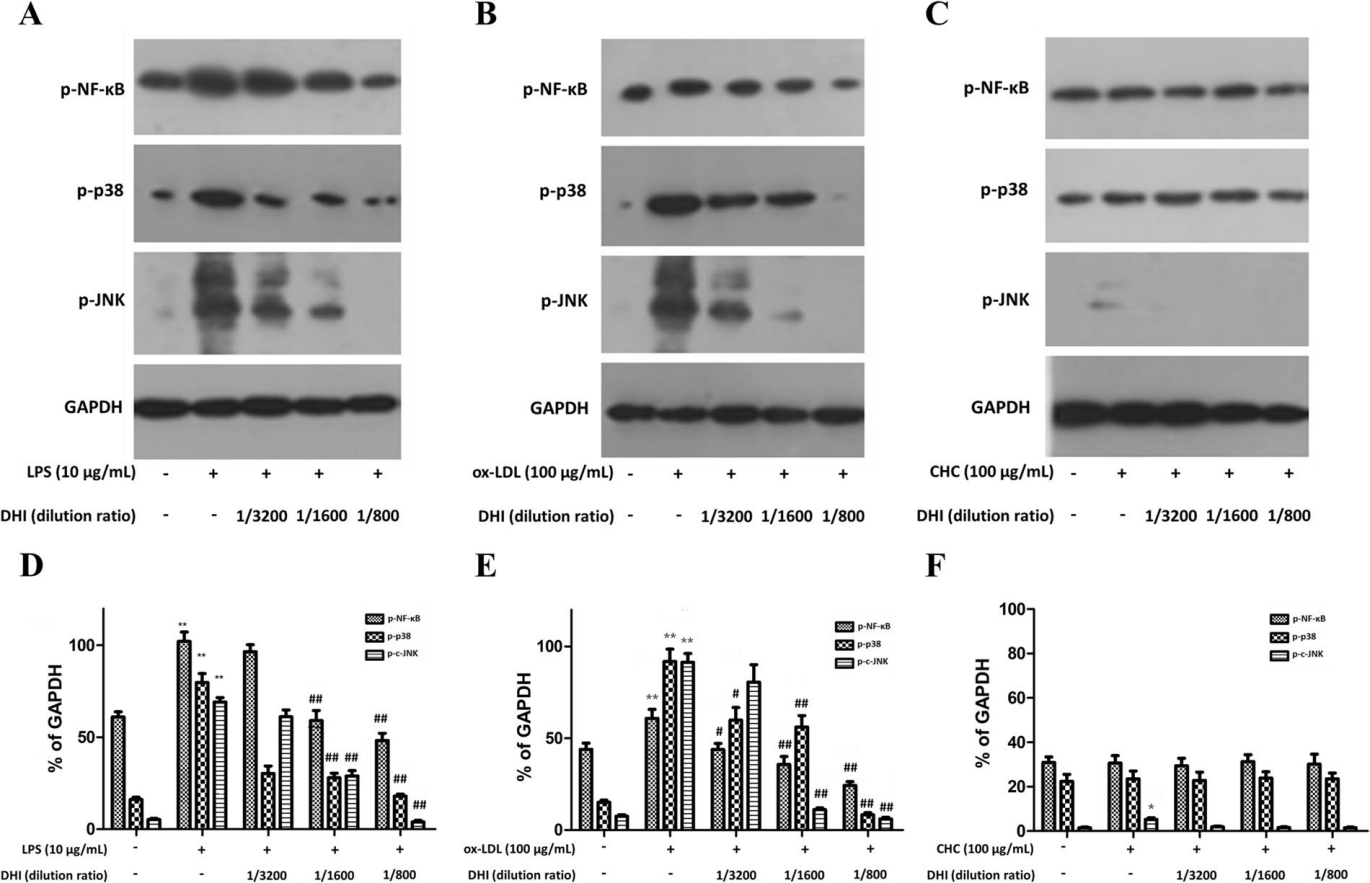
The effects of DHI on phosphorylation of NF-κB p65, p38, and JNK induced by different stimulants. Different concentrations of DHI were pre-incubated with EA.hy926 cells for 1 h. **(A**,**D)** DHI on 10 μg/mL LPS-induced phosphorylation of NF-κB, p38 and JNK. **(B**,**E)** DHI on 100 μg/mL ox-LDL-induced phosphorylation of NF-κB, p38 and JUK. **(C**,**F)** DHI on 100 μg/mL CHC-induced phosphorylation of NF-κB, p38 and JNK. Data are presented as mean ± SD (n = 3). *P < 0.05 versus control; **P < 0.01 versus control; ^#^P < 0.05 versus LPS or ox-LDL group; ^##^P < 0.01 versus LPS or ox-LDL group. Each Blot was cropped at the position of the blotted protein and high-contrast was not used.

To confirm the nuclear translocation results, phosphorylation level of JNK, p38 and NF-κB p65 were detected by Western blotting, which showed that JNK/c-Jun, p38, and NF-κB signalings were remarkably activated by 10 μg/mL LPS and 100 μg/mL ox-LDL **(**Fig. 6A,B,D and E **)**, but not by 100 μg/mL CHC except a slight up-regulation of p-JNK **(**Fig. 6E,F **)**. DHI with different dilution ratios (1/3200, 1/1600 and 1/800) dose-dependently suppressed 10 μg/mL LPS-induced phosphorylation of JNK, p38, and NF-κB p65 proteins **(**Fig. 6A,D **)**. Similarly, DHI with the same dilution ratios also dose-dependently suppressed 100 μg/mL ox-LDL-induced phosphorylation of JNK, p38 and NF-κB p65 proteins (Fig. 6B,E). Moreover, as 100 μg/mL CHC failed to stimulate p38 and NF-κB p65 phosphorylation but only slightly stimulated p-JNK phosphorylation up-regulation, the later effect was also abolished by DHI treatment (Fig. 6C,F). These Western blotting results were therefore largely consistent with the results of nuclear translocation.

### Effects of DHI on different stimulant-induced cytokines level of IL-1β, IL-10 and TNF-α

**Figure 7:**
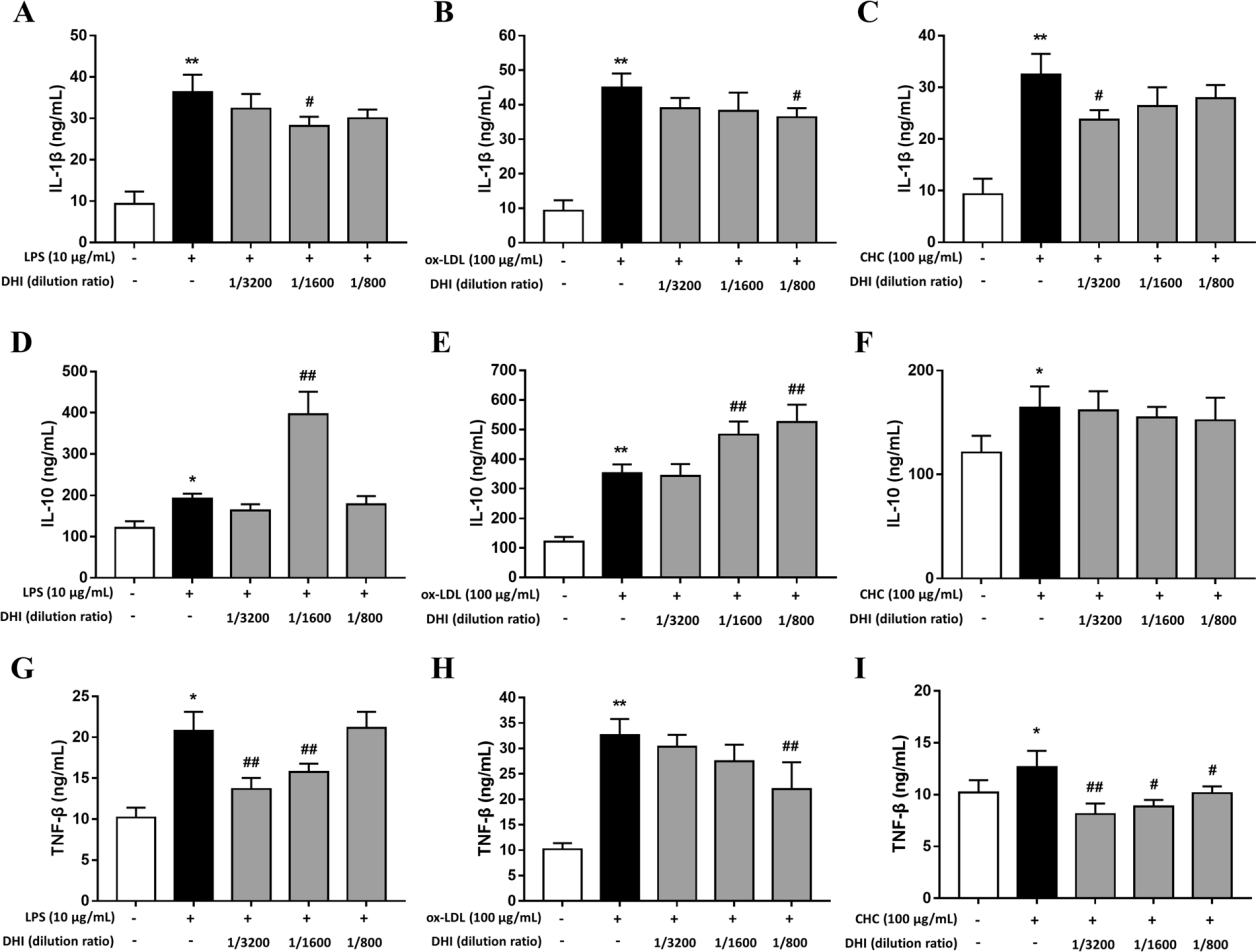
The effects of DHI on expression of IL-1β, IL-10 and TNF-α induced by different stimulants. EA.hy926 cells were pre-treated with different concentrations of DHI (1/800, 1/1600 and 1/3200 dilutions) for 1 h, and then stimulated with LPS, ox-LDL or CHC for 18 h respectively. Cell supernatants were collected to detect cytokines IL-1β, IL-10 and TNF-α levels. **(A–I)** IL-1β, IL-10 and TNF-α levels were promoted by 10 μg/mL LPS, 100 μg/mL ox-LDL and 100 μg/mL CHC. **(A–C)** DHI effects on LPS, ox-LDL or CHC-stimulated IL-1β level. **(D–F)** DHI effects on LPS, ox-LDL or CHC- stimulated IL-10 level. **(G–I)** DHI effect on LPS, ox-LDL or CHC-stimulated TNF-α level. Data are presented as mean ± SD (n = 3). *P < 0.05 versus control; **P < 0.01 versus control; ^#^P < 0.05 versus LPS, ox-LDL or CHC group; ^##^P < 0.01 versus LPS, ox-LDL or CHC group.

Based on IPA analysis shown above, the effects of DHI on the pro-inflammatory cytokines IL-1β, TNF-α and the anti-inflammatory cytokine IL-10 were determined in EA.hy926 cells. As expected, the levels of IL-1β, IL-10 and TNF-α were elevated by 10 μg/mL LPS, 100 μg/mL ox-LDL and 100 μg/mL CHC (Fig. 7). DHI was observed to attenuate LPS, ox-LDL and CHC-induced increase in the levels of IL-1β and TNF-α (Fig. 7A–C and G–I). In contract, DHI enhanced LPS and ox-LDL-induced IL-10 level (Fig. 7D,E). However, DHI had no significant effect on CHC-induced IL-10 level (Fig. 7F). Overall, DHI could decrease LPS, ox-LDL and CHC-induced pro-inflammatory IL-1β and TNF-α levels and reinforce LPS and ox-LDL-induced anti-inflammatory cytokines IL-10 level, but have no effect on CHC-induced IL-10 level.

### Validation of anti-inflammatory ingredients of DH by screening LPS-induced NF-κB p65 nuclear translocation

**Figure 8:**
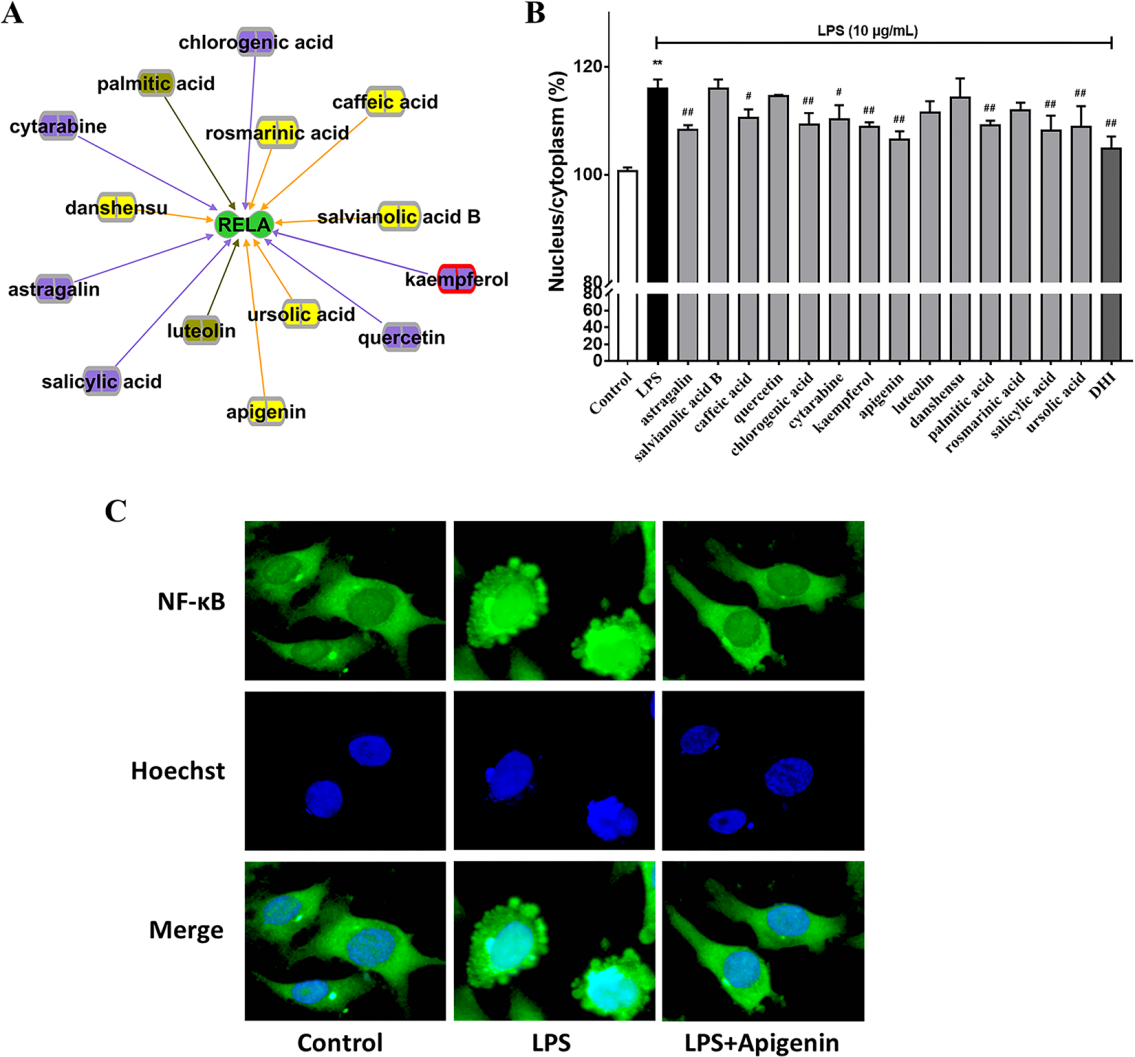
Anti-inflammatory screening of individual DH ingredients. **(A)** Sub-network of 14 ingredients predicted targeting NF-κB p65by network analysis. **(B)** EA.hy926 cells were cultured with each of the 14 DH ingredients (1 μM) for 1 h before adding 10 μg/mL LPS for 30 min. NF-κB p65 nuclear translocation assay was performed and quantification of the data was shown in bar graph. **(C)** Representative fluorescence microscopic images of apigenin effect on LPS-induced NF-κB p65 nuclear translocation. Nucleus were stained by Hoechst (blue) and the NF-κB p65 were stained by immunolabeled antibodies (green). Cells were imaged with the HCA reader using a 20× objective lens and then amplified to see the details, each column reflecting images collected from the respective fluorescent channels by the same optical field. Data are presented as mean ± SD (n = 3). **P < 0.01 versus control; ^#^P < 0.05 versus LPS group; ^##^P < 0.01 versus LPS group.

The sub-network of 14 ingredients (six from Danshen, six from Honghua and two shared by both) targeting NF-κB p65 (RELA) were extracted from the molecular network of Fig. 4D (Fig. 8A). HCA nuclear translocation assay was applied to validate the anti-inflammatory ingredients of DH in EA.hy926 cells. The percentage rate of NF-κB p65 nuclear translocation was enhanced by 10 μg/mL LPS. Compared with DHI (at 1/1600 dilutions), ten of the 14 ingredients (astragalin, salvianolic acid B, caffeic acid, chlorogenic acid, cytarabine, kaempferol, apigenin, palmitic acid, salicylic acid and ursolic acid) at 1 μM significantly inhibited LPS-induced NF-κB p65 nuclear translocation (Fig. 8B). A representative high-resolution fluorescence microscopic image of apigenin, one of the most potent compounds with its inhibitory effect on nuclear translocation of NF-κB p65, was exhibited (Fig. 8C). Data of the remaining 22 DH ingredients on NF-κB p65 nuclear translocation activity were shown in the Supplementary Fig. S3.

## Discussion

The novel findings of this study are: (1) by compound-target network analysis, we discover 37 potential active ingredients derived from DH are capable of modulating 68 common targets shared by stroke and CAD. (2) In target-disease & function-pathway network, mostly impacted diseases (atherosclerosis as #1), functions (inflammatory response as #1) and pathways (atherosclerosis signaling, HMGB1 signaling and LXR/RXR activation as #1–3) by DH are shared in stroke and CAD. (3) As a simplified model for DH, DHI exhibits a comprehensive anti-inflammatory effect on LPS, ox-LDL and CHC-induced endothelial inflammation, including NF-κB, c-jun, and p38 activation as well as IL-1β, TNF-α and IL-10 secretion, which indicate that anti-endothelial inflammation therapy may serve as a common underlying mechanism on both stroke and CAD treatment by DHI. (4) The anti-inflammatory ingredients predicted by the network analysis are validated in the model of LPS-induced endothelial inflammation. (5) Other top-ranked diseases and functions that are influenced by DH, such as dementia and Alzheimer’s disease, leukocyte migration, HMGB1 and HIF 1α signalings and LXR/RXR activation showed in Fig. 3, provide new clinical indications for DH and DHI that warrant future in-depth study.

An increasing number of studies had reported a close correlation between stroke and CAD. DHI had been widely applied in clinical treatment of both diseases. Both myocardial infarction area and cerebral infarction area were reduced by DHI in animal studies^41,55^. However, the shared underlying molecular mechanism of DHI on stroke and CAD protection remained unclear. Our network analysis suggested that inflammatory response and atherosclerosis were responsible for the potential shared comprehensive mechanisms of DHI in both stroke and CAD treatment. It is well recognized that inflammatory response and atherosclerosis are the principal pathogenic processes in ASCVD development and progression^21,90^. Leukocyte migration is important participants at the various stages of cardiovascular disease progression and complication^91^. Furthermore, inflammatory response regulates aspects of vascular biology that trigger the endothelial dysfunction and eventually leads to initiation of the plaque, formation of atherosclerotic lesions and their complications^92^.

Since there have been an explosive increase in our understanding of the molecular mechanisms of inflammation in recent years, we decided to make the *in vitro* cellular experiment more closely mimicking an *in vivo* inflammation setting and possibly distinguishing different molecular pathways. Therefore, we used three different inflammation triggers, LPS, ox-LDL and CHC. Interestingly, our data showed that cultured vascular endothelial cells responded to these inflammation triggers differently *in vitro*. While LPS and ox-LDL caused robust c-Jun, p38 or NF-κB p65 phosphorylation and nuclear translocation, CHC had no such effect (Figs 5 and 6). On the other hand, CHC significantly increased cytokine secretion similar to those of LPS and ox-LDL. Although the reason for this discrepancy is not presently clear, it is possible that vascular endothelial cells (VECs) lack a proper CHC receptor and/or CHC has to be converted/metabolized *in vivo*. It is worth noticing that DHI in most cases successfully reversed the phenotypes caused by all inflammatory triggers with one exception: it has significant effects on CHC-induced IL-1β and TNF-α secretion, but not on CHC-induced IL-10 secretion. One simplest possibility is that the CHC-induced IL-10 secretion was quite low and the assay sensitivity did not allow the detection of any DHI-mediated changes. Alternatively, one may hypothesize that CHC could enhance vascular wall inflammatory responses and lead to atherosclerosis by activeing NLRP3 inflammasomes, which regulate caspase-1 activation and subsequent processing of pro-IL-1β, triggering IL-1 secretion^38^, a processes inhabitable by DHI whereas the secretion of IL-10 is reduced in NLRP3−/− macrophage, which implying that NLRP3 inflammasomes activation may contribute to IL-10 secretion^93^, a process independent of DHI regulation. Further studies are needed to reveal the mechanism on how the activated inflammasomes regulate the secretion of IL-10 and DHI’s contribution in this process.

Our finding that endothelial inflammation as one of the most correlative mechanisms shared by DHI in the treatment of stroke and CAD is also supported by a number of observations from the bench to the bedside demonstrated that DHI exerted an anti-inflammatory effect^53^. In a recent clinical study, initial results from a randomized controlled trial suggested that DHI was effective in improving endothelial repair and protecting the endothelial lesion by mobilizing endothelial progenitor cells (EPCs) and inhibiting the inflammatory response after percutaneous coronary intervention in patients with CAD^52^. In patients with acute cerebral infarction, DHI was observed to lower the mRNA and protein levels of inflammatory cytokines (IL-6, TNF-α and IL-1β) and decreased nuclear NF-κB p65 expression in peripheral white blood cells^94^. Basic science studies identified at least nine potential anti-inflammatory ingredients in DHI: danshensu, protocatechuic acid, protocatechuic aldehyde, caffeic acid, hydroxysafflor yellow A, safflor yellow A, salvianolic acid A, salvianolic acid B and salvianolic acid C, which could significantly suppress inflammatory responses via TNF-α induced NF-κB pathway in EA.hy926 cells^53^. DHI could inhibit ox-LDL-induced maturation of dendritic cells partly through activating PPARγ-mediated signaling pathway^95^, which is confirmed by our upstream regulator analysis. DHI also exerted a protective effect through inhibiting the LPS-stimulated expressions of inducible NO synthase (iNOS), cyclooxygenase-2 (COX-2), IL-1β, IL-6, monocyte chemoattractant protein-1 (MCP-1) and TNF-α in macrophages^54,56^. Overall, DHI is capable of systematically inhibiting inflammatory response through multi-ingredient, multi-target and multi-pathway in both stroke and CAD related diseases. These previous reports further confirmed the accuracy of our network analysis. However, since stroke and CAD are two extraordinarily complicated diseases, our findings on DHI only covered a portion of the complex shared molecular mechanisms. Our network analysis also uncovers atherosclerosis and ApoE as the most relevant disease and upstream regulator, respectively, confirming that DHI is able to inhibit the development of atherosclerosis in ApoE^−/−^ mice^56^. Further in-depth investigation on DHI’s therapeutic effects is required to explore other identified critical functions such as leukocyte migration, angiogenesis and thrombosis, key pathways such as atherosclerosis signaling, HMGB1 signaling and LXR/RXR activation, important upstream regulators such as PPARG, TGFB1, IL6 and VEGFA, all of which had predicted by our network analysis.

Up till now, at least 63 compounds, including 33 phenolic acids, 2 C-glycosyl quinochalcones, 6 flavonoid O-glycosides, 4 iridoid glycosides, 6 organic acids, 5 amino acids, and 3 nucleosides were identified or tentatively characterized^62^. There is no doubt that far more potential active ingredients are to be detected in DHI. Only a few compounds of DHI were determined to exert definite pharmacological effects, including salvianolic acid B, danshensu, caffeic acid, rosmarinic acid, kaempferol, protocatechuic acid and hydroxysafflor yellow A^51,53,54,58,62,63^, which were highlighted with red border in the network (Fig. 3A). These limited compouds were certainly insufficient to explain the entire pharmacological functions of DHI. Consequently, it remained a great challenge to reveal the molecular mechanism of a complex formula at a systematic level. To our delight, over the last 50 years, the chemical constituents and biological activities of Danshen and Honghua have been well studied. More than 100 compounds have been isolated and identified from each of them^96–98^. A growing number of TCM platforms, such as TCMID^99^ and TCMSP^100^ had been established for integrative relationships between herbs and their treated diseases, as well as the active ingredients and their targets. They will facilitate the study of combination therapy and our understanding of the underlying mechanisms for TCM at molecular level. To overcome these problems, the ingredients from Danshen and Honghua were taken into account, contributing to investigating the system-pharmacology mechanism of DHI. However, this bold attempt is a double-edged sword. On one hand, we can understand DHI more comprehensive and discover more potential ingredients, which has not been identified or reported. On the other hand, the analysis results which we achieved are inescapability broader than DHI possessed. On all accounts, all of the prediction outcomes should be validated by experimental vetification.

Despite of abundant new findings in this study, some limitations still exist. The following directions and prospects should be considered in future investigation: (1) The accuracy of a network pharmacology analysis of a complex system such as a compound Chinese medicine (CCM) is critically dependent on the resolution of its chemical basis. Conventional LC-MS approach was confronted with increasing challenges arising from limited peak capacity and selectivity, which are also a common issue that impedes elucidation of the therapeutic basis for most herbal medicines as well as their products. DHI is one of the simplest and chemically best resolved CCM. We and others have introduced new techniques such as ^1^H NMR to identify and quantify additional ingredients^62,64^. More advanced ingredient identification method, such as 2D LC/QTOF, should be applied to identify more ingredients in DHI, which will benefit our understanding of its therapeutic effects^101^. (2) Based on the 68 common targets, we also performed the PPI network to find the node targets (see supplement Figure S4). This is a conventional method to obtain the key targets. Most of the nodes obtained by PPI were in accordance with the top upstream regulators analysis used in our study. The principle of the former is based on the interaction between protein and protein, whereas the latter depends on the upstream and downstream regulatory effects by incorporating different pathways. Both are effective means to discover the targets of interest. Which is better in the application of TCM network analysis need to be further explored. (3) Although our immunofluorescence and Western blot data showed that DHI had a good curative effect in a dose-dependent manner, the ELISA data only exhibited a partial dose independence. This maybe owing to the stability of experimental system and complicacy of TCM. In addition, DHI has significant effects on CHC-induced IL-1β and TNF-α secretion, but has no significant effect only on CHC-induced IL-10 secretion. Based on this phenomenon, we have one hypothesis to interpret it. For one thing, CHC can enhance vascular wall inflammatory responses and lead to atherosclerosis by active NLRP3 inflammasomes, which regulate caspase-1 activation and subsequent processing of pro-IL-1β, triggering IL-1 secretion^38,102^. These processes are able to be inhibited by DHI. On the other hand, the secretion of IL-10 is reduced in NLRP3−/− macrophage, which implying that NLRP3 inflammasomes activation may contribute to IL-10 secretion^94^. This process can’t be regulated by DHI. The mechanism on how the activated inflammasomes regulate the secretion of IL-10 is lacking, which need to be further studied. Notwithstanding, DHI still showed a benefit effect on different stimulants-induced inflammatory cytokines secretion. (4) More active anti-inflammatory ingredients should be verified in different inflammatory models. (5) Multiple omics approaches should be exploited *in vivo* to complement and confirm the results. For example, DHI-treated animal samples from stroke or CAD disease models could be collected, and proteomics or transcriptome analysis be performed to get the overlapping targets.

In conclusion, a comprehensive approach integrating network pharmacology analysis and experimental validation was taken for the first time to systematically investigate the key common targets and action mechanisms of DHI for stroke and CAD. The ingredient-target-disease-function-pathway network revealed that atherosclerosis and endothelial inflamation are the most critical action targets by DHI for both stroke and CAD treatment. Our findings may shed a new light on the mechanisim of co-treatment of stroke and CAD by a multi-targeting anti-inflamatory agent such as DHI and its potential in clinical application.

## Materials and Methods

### Database construction

The main source of disease targets for both stroke and CAD were obtained from IPA (http://www.ingenuity.com) database^87,103^. Additional databases such as OMIM^104^, CADGene^105^, NCBI-gene, GeneCards^106^ and MalaCards^107^ were manually searched and information subtracted to complement the omissions of IPA. Duplicate genes were removed by screening. Information on DH ingredients were retrieved from several TCM datasets, including TCMSP^100^, TCM Database@Taiwan^108^, TCM-ID^109^, TCMGeneDIT^110^ and literature mining^51,53,54,58,62,63,96,97,111^. As a compound is often represented by more than one chemical name, we discerned them by molecular structure and then transferred them into PubChem CID^112^ or CAS number which IPA software could recognize. TCMSP^100^ and TCMID^99^ were also employed to replenish DHI ingredients corresponding targets which IPA database may not record. In brief, the database of DH major ingredients and stroke and CAD related targets was constructed through searching IPA along with certain TCM and bioinformatics websites. Details including web links to the databases were shown in Supplementary Table S1.

### Network establishment and analysis

Three datasets, including ①Danshen and Honghua ingredients, ②stroke and CAD associated targets, and ③DHI’s major ingredients and their corresponding targets, were constructed and then uploaded into the IPA system to enable the discovery visualization. “Build-Path Explorer” module was applied to discover stroke- and CAD-related targets, and the relationship between DH ingredients and the targets. “Build-Connection” module was implemented to interpret the relationship between targets and targets. “Overlay-Canonical Pathway” module was used to generate the resulting canonical pathways. “Build-Diseases & Functions” module was exploited to build the targets involved diseases and functions. Finally, an integrated compound-target-pathway-disease & function network was constructed. We utilized “Core analysis” module to analyze the correlation degree of the network which we established before, so that we could acquire top diseases, top functions, top pathways and top upstream regulators. Upstream regulators analyses were aimed at elucidating the causal inference of upstream biological causes and probable downstream effects on cellular and organismal biology^103^. This application was similar with the approach taken by the Connectivity Map tool^113^. Certain top upstream regulators which we concerned were defined by “Upstream Regulator” module. “Path designer” module was performed to clarify and beautify the network. In this study, the algorithm of the network analysis was based on Fisher’s exact test with the enrichment score of P-values.

### Drugs and Regents

DHI was kindly donated by Shandong BuChang Pharmaceutical Co., Ltd. (Jinan, China, drug approval number: Z20026866). Lipopolysaccharide (LPS), Cholesterol were purchased from Sigma (MO, USA). Cholesterol crystals (CHC) were produced as previously described^37^. Ox-LDL was purchased from Yiyuan biotechnology (Guangzhou, China). Dulbecco’s modified Eagle’s medium (DMEM), fetal bovine serum (FBS), L-glutamine, penicillin, and streptomycin were purchased from Gibco (NY, USA). Mouse anti-p-c-Jun [Ser63] antibody, goat anti-p-NF-κB p65 antibody, goat anti-NF-κB p65 antibody, goat anti-mouse IgG-HRP and goat anti-rabbit IgG-HRP were purchased from Santa Cruz Biotechnology, Inc (Santa Cruz, CA). Rabbit anti-p-p38 [Thr180/Tyr182] antibody and mouse anti-p-JNK [Thr183/Tyr185] were purchased from Cell Signaling Technology (MA, USA). Donkey Anti-Mouse IgG H&L (Alexa Fluor® 488), Donkey Anti-Rabbit IgG H&L (Alexa Fluor® 555), Donkey Anti-Goat IgG H&L (Alexa Fluor® 647) and Goat Anti-Rabbit IgG H&L (Alexa Fluor® 488) were obtained from Abcam (UK). Triton X-100, 4% paraformaldehyde, and bovine serum albumin (BSA) were purchased from Solarbio (Beijing, China), and Hoechst 33342 was purchased from Invitrogen (CA, USA). Cell Counting Kit-8 (CCK-8) was produced by Dojindo Laboratories (Tokyo, Japan). Cytokine IL-1β, IL-10 and TNF-α ELLISA kits were obtained from Wuhan antgene Biotechnology Co., Ltd. (Wuhan, China). Compounds were brought from ChengduPush Bio-technology Co., Ltd (Chengdu, China).

### Cell viability

EA.hy926 cells were purchased from Cell Bank of the Chinese Academy of Sciences (Shanghai, China) and maintained in high-glucose DMEM supplemented with 10% FBS, Hyclone, L-glutamine (2 mM), 100 units/mL of penicillin and 100 μg/mL streptomycin. Cells were incubated in a humidified incubator aerated with 5% CO_2_ at 37 °C. DMEM with 10% FBS in was replaced to serum-free medium when cells were grown to approximately 70% to 80% confluences. The effect of DHI on cell viability was evaluated using CCK-8 kit. In brief, EA.hy926 cells were seeded in 96-well plate at a density of 1.5 × 10^4^ cells/well and incubated at 37 °C for 24 h. Then, the cells were treated with various concentrations of DHI. After 24 h incubation, 10 μL CCK-8 solution was added to the wells, and continued for another 3 h incubation. The resulting color was assayed at 450 nm using FlexStation® 3 (Molecular Devices, Emax, Sunnyvale, CA).

### Immunofluorescence and Western blot assay

EA.hy926 cells were cultured in high-glucose DMEM supplemented with 10% FBS, L-glutamine (2 mM), 100 units/mL of penicillin and 100 μg/mL streptomycin. Then, the cells were subcultured in black optically clear-bottomed 96-well Packard ViewPlates™ plate (PerkinElmer, MA, USA) at 1.5 × 10^4^ cells/well for 24 h. Following incubation with different concentrations of LPS, ox-LDL or CHC in serum-free DMEM for 30 min, cells were fixed by 4% paraformaldehyde at room temperature and washed three times with 200 μL of PBS by flicking off the wash buffer and gently tapping the plate on tissues. The cells were permeabilized with 0.2% Triton X-100 for 5 min and rinsed once with PBS. Non-specific binding sites were blocked by incubation with 2% BSA for 1 h prior to incubation with primary antibody overnight. The mixture of primary antibody containing mouse anti-p-c-Jun antibody (1:200), rabbit anti-p-p38 antibody (1:300) and goat anti-p-NF-κB p65 (1:200) antibodies in PBS supplemented with 0.01% Tween 20 and 0.2% BSA. Following primary antibody incubation, cells were washed in PBS containing 0.05% Tween 20 for 5 min and rinsed twice in PBS before incubation with secondary antibodies of donkey anti-mouse IgG H&L (1:200), donkey anti-Rabbit IgG H&L (1:200) and donkey anti-goat IgG H&L (1:200), along with 0.625 μg/mL Hoechst 33342 to fluorescently label cell nuclei. Cells were again washed in 200 μL PBS containing 0.05% Tween 20 for 5 min on a shaker device and rinsed twice in 200 μL PBS. The final washing PBS was left in the wells and the plate was sealed. Plate was scanned by HCA (PerkinElmer, MA, USA) and then Harmony 3.0 software was used to calculate the percentage rate of nuclear translocation^114^. At the end of the experiment, 10 μg/mL LPS, 100 μg/mL ox-LDL and 100 μg/mL CHC were selected as final stimulus concentrations in the following experiments. Cells were pre-incubated with DHI for 1 h before addition of 10 μg/mL LPS or 100 μg/mL ox-LDL for 30 min, followed by immunofluorescence as described earlier.

To determine anti-inflammatory effects of DHI through JNK/c-Jun, P38 and NF-κB signaling, protein phosphorylation was measured by Western blotting assay. Briefly, equal amounts of 50 μg protein extracts were separated by 12% SDS-polyacrylamide gels, and then transferred onto polyvinylidene fluoride (PVDF) membrane (Millipore, USA). The membrane was blocked with 5% BSA in TBST for 1.5 h at room temperature, and then incubated over-night at 4 °C with the primary antibodies of mouse anti-p-JNK (1:1000), rabbit anti-p-p38 (1:1000) and goat anti-p-NF-κB p65 (1:1000). After washing three times with TBST, the membrane was incubated for 1 h with goat anti-mouse IgG-HRP (1:1000) and goat anti-rabbit IgG-HRP (1:1000) as the secondary antibody at room temperature. After washing three times, the immunoblots were detected by enhanced chemiluminescence (ECL) detection kit (CoWin Biotech Co., Ltd., Beijing, China). GAPDH was used as endogenous control. Data were normalized to GAPDH levels. The quantification of bands was performed according to densitometry by ImageJ software.

### Enzyme-linked immunosorbent assay (ELISA)

For the determination of the expression levels of IL-1β, IL-10 and TNF-α, the EA.hy926 cells were treated with different concentrations of DHI for 1 h and then stimulated with 10 μg/mL LPS, 100 μg/mL ox-LDL and 100 μg/mL CHC for 18 h. The supernatants of cells were analyzed using a human IL-1β, IL-10 or TNF-α ELISA kit according to the manufacturer’s instructions. In brief, standards and samples were diluted with PBS, loaded onto a 96-well plate and incubated for 90 min at 37 °C. Next, biotin-labeled antibodies against IL-1β, IL-10 and TNF-α were utilized for specific binding. Finally, an avidin-labeled enzyme and substrate were used to quantify the levels of IL-1β, IL-10 and TNF-α using FlexStation® 3 (Molecular Devices, Sunnyvale, CA). The concentration of IL-1β, IL-10 and TNF-α were calculated with the reference to the standard curve obtained from a standard solution provided by the kit.

### Active ingredients screening

To verify the anti-inflammatory activity of the DH ingredients predicted by our network analysis, LPS-induced NF-κB p65 nuclear translocation assay was performed as described above. In brief, EA.hy926 cells were cultured in the presence or absence of the 36 individual ingredients (at 1 μM each) for 1 h before adding 10 μg/mL LPS for 30 min. Primary rabbit anti-NF-κB p65 antibody (1:200) and second goat anti-rabbit IgG H&L antibody (Alexa Fluor® 488) were sequentially added in wells. HCA and Harmony 3.0 software were utilized to collect and analyze data.

### Data analysis

All experiments were repeated at least 3 times and presented as mean ± SD and analyzed by one-way ANOVA. P < 0.05 was considered to indicate a statistically significant difference. All tests were performed using GraphPad Prism 7 software (GraphPad Software, Inc., La Jolla, CA, USA).

### Data availability

The datasets generated during and/or analyzed in the current study are available from the corresponding author upon request.

## Electronic supplementary material

Supplementary dataset 1
