# Supplementary material for: Network pharmacology exploration reveals endothelial inflammation as a common mechanism for stroke and coronary artery disease treatment of Danhong injection
Source: Sci Rep. 2017 Nov 13;7:15427. doi: 10.1038/s41598-017-14692-3 (PMC5684234; doi:10.1038/s41598-017-14692-3)
Supplement: Supplementary file 1 — Supplementary dataset 1 [file 41598_2017_14692_MOESM1_ESM.docx]

**Network pharmacology exploration reveals endothelial inflammation as a common mechanism for stroke and coronary artery disease treatment of Danhong injection**

Ming Lu^1,2+^, Chun-Lin Yan^1,2+^, Hai-Xin Liu^1,2^, Tai-Yi Wang^1,2^, Xin-Hui Shi^1,2^, Jin-Ping Liu^1,2^, John Orgah^1,2^, Guan-Wei Fan^1,2,3^, Ji-Hong Han^4^, Xiao-Ying Wang^1,5^, Yan Zhu^1,2,6*^

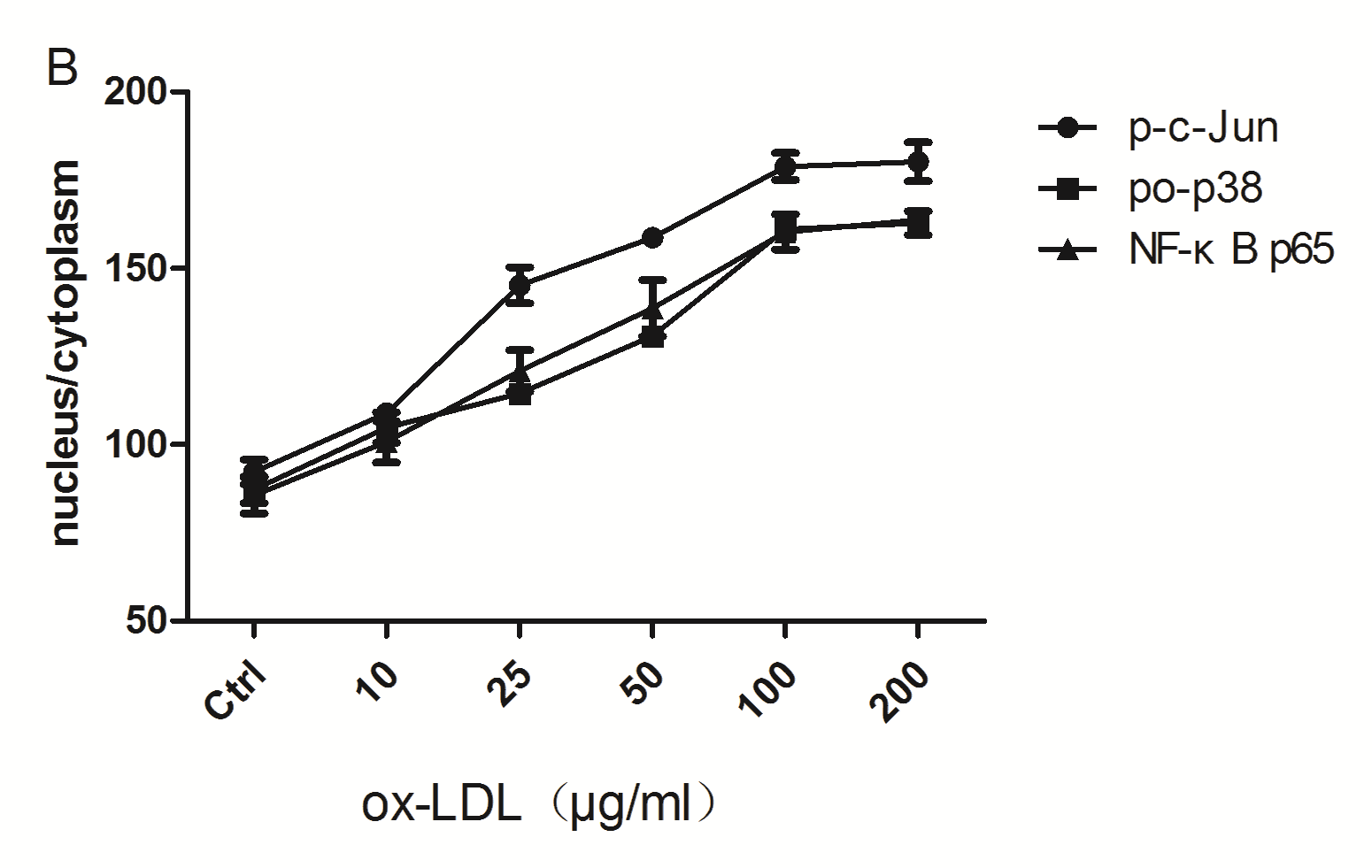


**(%)**

**Supplementary Figure S1. Cell activity assay.** Cell viability is scarcely affect by DHI at the ratio of 1/3200, 1/1600 and 1/800. Cell viability was affected by DHI when dilutes into 1/200 and 1/100. Data are presented as mean ± SD (n=3). **P＜0.01 versus control.


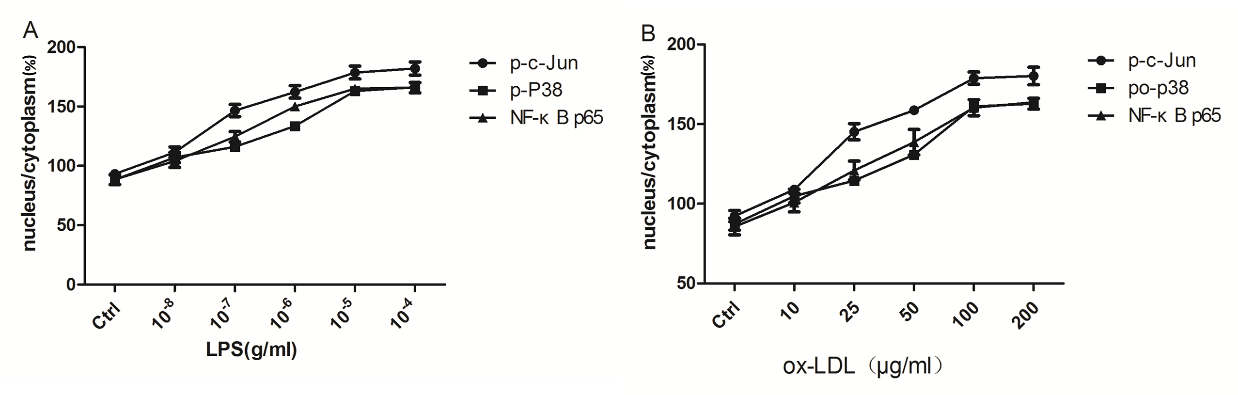


**Supplementary Figure S2. LPS and ox-LDL induced nuclear translocation.** **(A)** LPS and **(B)** ox-LDL stimulate nuclear translocation of p-c-Jun, p-p38 MAPK, p-NF-κB p65 in a dose-dependent manner. Data are presented as mean ± SD (n=3).

**Supplementary Figure S3. The rest 22 DH ingredients anti-inflammatory effect screening.** Rutin, catechol, baicalein, myricetin and ferulic acid could significantly inhibit LPS induced NF-κB p65 nuclear translocation. Data are presented as mean±SD (n = 3). **P＜0.01 versus control; # P＜0.05 versus LPS group; ## P＜0.01 versus LPS group.


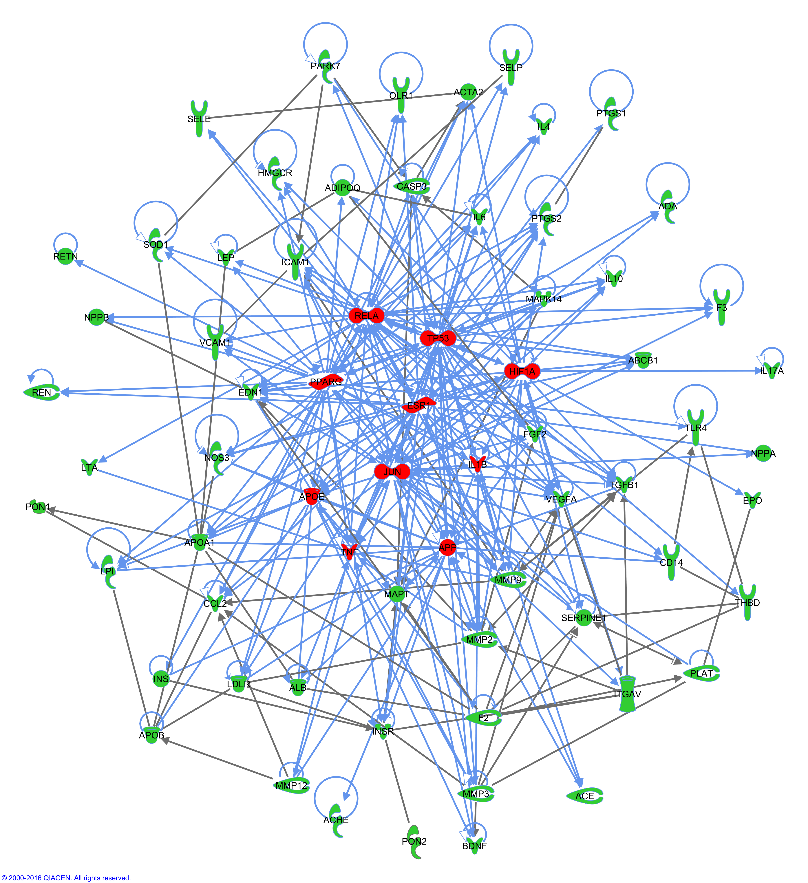


**Supplementary Figure S4. Protein-protein interaction network of the 68 common targets.**

| Category | **Databases** | **Website** | **References** |
| --- | --- | --- | --- |
| Stroke and CAD targets | IPA | http://www.ingenuity.com/ | [^1^](#_ENREF_1) |
|  | OMIM | http://omim.org/ | [^2^](#_ENREF_2) |
|  | CADGene | http://www.bioguo.org/CADgene/ | [^3^](#_ENREF_3) |
|  | NCBI (gene) | http://www.ncbi.nlm.nih.gov/gene/ | [^4^](#_ENREF_4) |
|  | GeneCards | http://www.genecards.org/ | [^5^](#_ENREF_5) |
|  | MalaCards | http://www.malacards.org/ | [^6^](#_ENREF_6) |
| DH (Danshen, Honghua  and DHI) ingredients | TCMSP | http://lsp.nwsuaf.edu.cn/tcmsp.php | [^7^](#_ENREF_7) |
|  | TCM Database@Taiwan | http://tcm.cmu.edu.tw/ | [^8^](#_ENREF_8) |
|  | TCMID | http://www.megabionet.org/tcmid/ | [^9^](#_ENREF_9) |
|  | TCM-ID | http://tcm.cz3.nus.edu.sg/group/tcm-id/tcmid_ns.asp | [^10^](#_ENREF_10) |
|  | TCMGeneDIT | http://tcm.lifescience.ntu.edu.tw/ | [^11^](#_ENREF_11) |
|  | PubMed | http://www.ncbi.nlm.nih.gov/pubmed/ | [^12-20^](#_ENREF_12) |
| DHI major ingredients  corresponding targets | IPA | http://www.ingenuity.com/ | [^1^](#_ENREF_1) |
|  | TCMID | http://www.megabionet.org/tcmid/ | [^9^](#_ENREF_9) |
|  | TCMSP | http://lsp.nwsuaf.edu.cn/tcmsp.php | [^7^](#_ENREF_7) |

**Supplementary Table S1 Details of DH Ingredients and diseases associated databases**

| **No.** | **Symbol** | **Entrez Gene Name** | **Entrez Gene ID for Human** | **Entrez Gene ID for Mouse** | **Entrez Gene ID for Rat** | **Drugs** |
| --- | --- | --- | --- | --- | --- | --- |
| 1 | ABCB1 | ATP binding cassette subfamily B member 1 | 5243 | 18671 | 170913 | dofequidar, tariquidar, OC 144-093, valspodar |
| 2 | ACE | angiotensin I converting enzyme | 1636 | 11421 | 24310 | pentopril, quinaprilat, perindoprilat, amlodipine/perindopril, benazeprilat |
| 3 | ACHE | acetylcholinesterase (Cartwright blood group) | 43 | 11423 | 83817 | ZT-1, ladostigil, terestigmine, acetylcholinesterase inhibitor, donepezil/memantine |
| 4 | ACTA2 | actin, alpha 2, smooth muscle, aorta | 59 | 11475 | 81633 |  |
| 5 | ADA | adenosine deaminase | 100 | 11486 | 24165 | pentostatin, aspirin/dipyridamole/telmisartan, cyclophosphamide/pentostatin/rituximab, pentostatin/rituximab, alemtuzumab/pentostatin |
| 6 | ADIPOQ | adiponectin, C1Q and collagen domain containing | 9370 | 11450 | 246253 |  |
| 7 | ALB | albumin | 213 | 11657 | 24186 | gadofosveset trisodium, aldoxorubicin |
| 8 | APOA1 | apolipoprotein A1 | 335 | 11806 | 25081 |  |
| 9 | APOB | apolipoprotein B | 338 | 238055 | 54225 | mipomersen |
| 10 | APOE | apolipoprotein E | 348 | 11816 | 25728 |  |
| 11 | APP | amyloid beta precursor protein | 351 | 11820 | 54226 | bapineuzumab, florbetapir F18, florbetaben F |
| 12 | BDNF | brain derived neurotrophic factor | 627 | 12064 | 24225 |  |
| 13 | CASP3 | caspase 3 | 836 | 12367 | 25402 | caspase 3 inhibitor, emricasan |
| 14 | CCL2 | C-C motif chemokine ligand 2 | 6347 | 20293 | 287562 | mimosine |
| 15 | CD14 | CD14 molecule | 929 | 12475 | 60350 |  |
| 16 | EDN1 | endothelin 1 | 1906 | 13614 | 24323 |  |
| 17 | EPO | erythropoietin | 2056 | 13856 | 24335 |  |
| 18 | ESR1 | estrogen receptor 1 | 2099 | 13982 | 24890 | 17-alpha-ethinylestradiol, fulvestrant, beta-estradiol, estradiol 17beta-cypionate, estriol |
| 19 | F2 | coagulation factor II, thrombin | 2147 | 14061 | 29251 | enoxaparin, desirudin, dabigatran etexilate, ximelagatran, thrombin inhibitor, aspirin/dabigatran etexilate, aspirin/bivalirudin, bivalirudin |
| 20 | F3 | coagulation factor III, tissue factor | 2152 | 14066 | 25584 | activated recombinant human factor VII |
| 21 | FGF2 | fibroblast growth factor 2 | 2247 | 14173 | 54250 | pentosan polysulfate, suradista, CP-547632, sucralfate |
| 22 | HIF1A | hypoxia inducible factor 1 alpha subunit | 3091 | 15251 | 29560 | EZN 2968 |
| 23 | HMGCR | 3-hydroxy-3-methylglutaryl-CoA reductase | 3156 | 15357 | 25675 | aspirin/pravastatin, beta-hydroxy simvastatin acid, ezetimibe/fluvastatin, atorvastatin/niacin, fenofibric acid/rosuvastatin |
| 24 | ICAM1 | intercellular adhesion molecule 1 | 3383 | 15894 | 25464 |  |
| 25 | IL10 | interleukin 10 | 3586 | 16153 | 25325 |  |
| 26 | IL17A | interleukin 17A | 3605 | 16171 | 301289 | secukinumab, ixekizumab |
| 27 | IL1B | interleukin 1 beta | 3553 | 16176 | 24494 | canakinumab, gevokizumab, canakinumab/INS, gallium nitrate |
| 28 | IL4 | interleukin 4 | 3565 | 16189 | 287287 |  |
| 29 | IL6 | interleukin 6 | 3569 | 16193 | 24498 | tocilizumab, siltuximab |
| 30 | INS | insulin | 3630 | 16334 | 24506 |  |
| 31 | INSR | insulin receptor | 3643 | 16337 | 24954 | BMS-754807, insulin detemir, INS, canakinumab/INS, insulin aspart/insulin detemir, ceritinib |
| 32 | ITGAV | integrin subunit alpha V | 3685 | 16410 | 296456 | abciximab, CNTO 95, cilengitide |
| 33 | JUN | Jun proto-oncogene, AP-1 transcription factor subunit | 3725 | 16476 | 24516 |  |
| 34 | LDLR | low density lipoprotein receptor | 3949 | 16835 | 300438 |  |
| 35 | LEP | leptin | 3952 | 16846 | 25608 |  |
| 36 | LPL | lipoprotein lipase | 4023 | 16956 | 24539 | atorvastatin/niacin, nicotinic acid/pioglitazone, nicotinic acid, tyloxapol, lovastatin/niacin |
| 37 | LTA | lymphotoxin alpha | 4049 | 16992 | 25008 | etanercept, etanercept/methotrexate |
| 38 | MAPK14 | mitogen-activated protein kinase 14 | 1432 | 26416 | 81649 | talmapimod, RO-3201195, ralimetinib, p38 MAP kinase inhibitor |
| 39 | MAPT | microtubule associated protein tau | 4137 | 17762 | 29477 |  |
| 40 | MMP12 | matrix metallopeptidase 12 | 4321 | 17381 | 117033 | marimastat |
| 41 | MMP2 | matrix metallopeptidase 2 | 4313 | 17390 | 81686 | MMP2 MMP9 inhibitor, rebimastat, marimastat, prinomastat |
| 42 | MMP3 | matrix metallopeptidase 3 | 4314 | 17392 | 171045 | marimastat |
| 43 | MMP9 | matrix metallopeptidase 9 | 4318 | 17395 | 81687 | MMP2 MMP9 inhibitor, GS-5745, rebimastat, marimastat, prinomastat, glucosamine |
| 44 | NOS3 | nitric oxide synthase 3 | 4846 | 18127 | 24600 | 5,6,7,8-tetrahydrobiopterin, GW 273629, targinine |
| 45 | NPPA | natriuretic peptide A | 4878 | 230899 | 24602 |  |
| 46 | NPPB | natriuretic peptide B | 4879 |  |  |  |
| 47 | OLR1 | oxidized low density lipoprotein receptor 1 | 4973 | 108078 | 140914 |  |
| 48 | PARK7 | Parkinsonism associated deglycase | 11315 | 57320 | 117287 |  |
| 49 | PLAT | plasminogen activator, tissue type | 5327 | 18791 | 25692 | 6-aminocaproic acid |
| 50 | PON1 | paraoxonase 1 | 5444 | 18979 | 84024 |  |
| 51 | PON2 | paraoxonase 2 | 5445 | 330260 | 296851 |  |
| 52 | PPARG | peroxisome proliferator activated receptor gamma | 5468 | 19016 | 25664 | icosapent, amlodipine/telmisartan, inolitazone, aleglitazar, aspirin/dipyridamole/telmisartan, clopidogrel/telmisartan |
| 53 | PTGS1 | prostaglandin-endoperoxide synthase 1 | 5742 | 19224 | 24693 | acetaminophen/pentazocine, acetaminophen/clemastine/pseudoephedrine, aspirin/butalbital/caffeine, acetaminophen/caffeine/dihydrocodeine, aspirin/hydrocodone, aspirin/oxycodone |
| 54 | PTGS2 | prostaglandin-endoperoxide synthase 2 | 5743 | 19225 | 29527 | acetaminophen/pentazocine, aspirin/butalbital/caffeine, acetaminophen/caffeine/dihydrocodeine, aspirin/hydrocodone, aspirin/oxycodone, aspirin/omeprazole, COX2 inhibitor |
| 55 | RELA | RELA proto-oncogene, NF-kB subunit | 5970 | 19697 | 309165 | NF-kappaB decoy |
| 56 | REN | renin | 5972 | 19701 | 24715 | aliskiren, aliskiren/valsartan, aliskiren/amlodipine, aliskiren/amlodipine/hydrochlorothiazide, remikiren |
| 57 | RETN | resistin | 56729 | 57264 | 246250 |  |
| 58 | SELE | selectin E | 6401 | 20339 | 25544 |  |
| 59 | SELP | selectin P | 6403 | 20344 | 25651 |  |
| 60 | SERPINE1 | serpin family E member 1 | 5054 | 18787 | 24617 | drotrecogin alfa |
| 61 | SOD1 | superoxide dismutase 1, soluble | 6647 | 20655 | 24786 |  |
| 62 | TGFB1 | transforming growth factor beta 1 | 7040 | 21803 | 59086 | dalantercept |
| 63 | THBD | thrombomodulin | 7056 | 21824 | 83580 |  |
| 64 | TLR4 | toll like receptor 4 | 7099 | 21898 | 29260 | resatorvid, OM 174 lipid |
| 65 | TNF | tumor necrosis factor | 7124 | 21926 | 24835 | adalimumab, etanercept, infliximab, certolizumab, golimumab, infliximab/methotrexate, dexamethasone/thalidomide |
| 66 | TP53 | tumor protein p53 | 7157 | 22059 | 24842 | APR-246, cenersen, CGM097, kevetrin, azurin 50-77 |
| 67 | VCAM1 | vascular cell adhesion molecule 1 | 7412 | 22329 | 25361 |  |
| 68 | VEGFA | vascular endothelial growth factor A | 7422 | 22339 | 83785 | dalteparin, bevacizumab, ranibizumab, aflibercept, bevacizumab/erlotinib, bevacizumab/sorafenib, bevacizumab/5-fluorouracil, bevacizumab/temozolomide |

**Supplementary Table S2 Details of 68 common potential targets**

| **Herb** | **Ingredients** | **Stucture** | **OB (%)** | **Caco-2** | **BBB** | **DL** | **LogP** |
| --- | --- | --- | --- | --- | --- | --- | --- |
| Danshen | alpha-tocopherol | 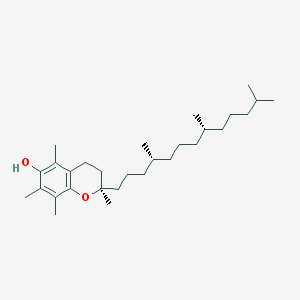 | 14.26 | 1.7 | 1.67 | 0.55 | 10.42 |
| Danshen | 3,4-dihydroxybenzaldehyde | 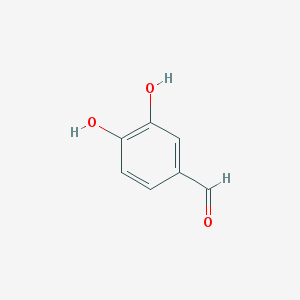 | 38.35 | 0.43 | 0.21 | 0.03 | 1.06 |
| Danshen | apigenin | 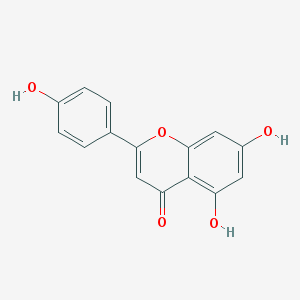 | 23.06 | 0.43 | -0.61 | 0.21 | 2.33 |
| Danshen | caffeic acid | 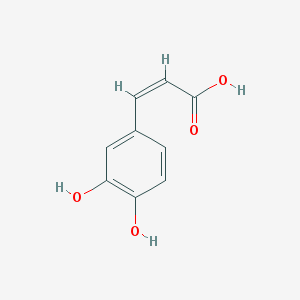 | 54.97 | 0.27 | 0.11 | 0.05 | 1.37 |
| Honghua | astragalin | 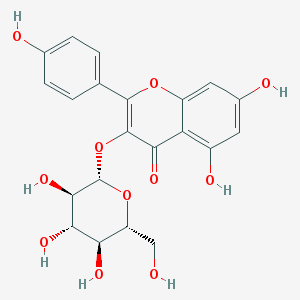 | 14.03 | -1.34 | -1.97 | 0.74 | -0.32 |
| Honghua | baicalein | 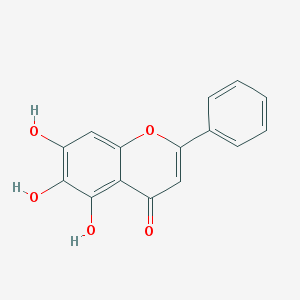 | 33.52 | 0.63 | -0.05 | 0.21 | 2.33 |
| Common | baicalin | 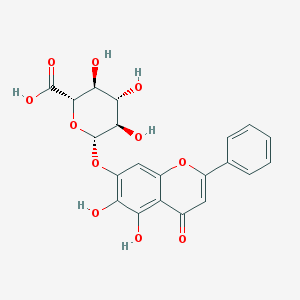 | 40.12 | -0.85 | -1.74 | 0.75 | 0.64 |
| Danshen | carnosol | 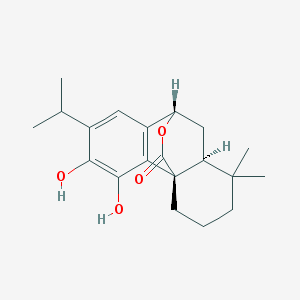 | 14.96 | 0.80 | 0.56 | 0.43 | 4.34 |
| Honghua | carvacrol | 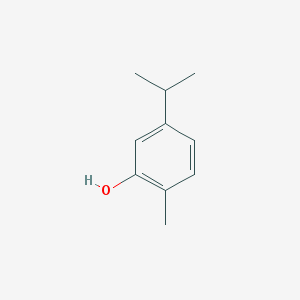 | 43.28 | 1.58 | 1.71 | 0.03 | 3.24 |
| Honghua | catechol | 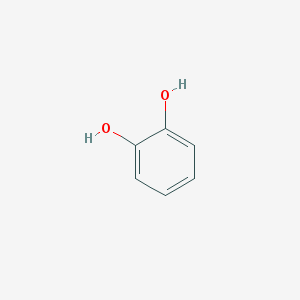 | 29.86 | 1.09 | 1.32 | 0.02 | 1.30 |
| Honghua | chlorogenic acid | 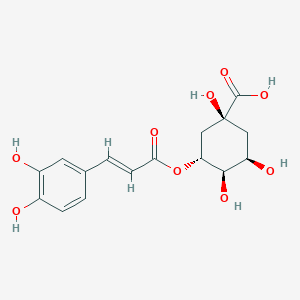 | 13.61 | -1.33 | -1.79 | 0.31 | -0.27 |
| Danshen | cryptotanshinone | 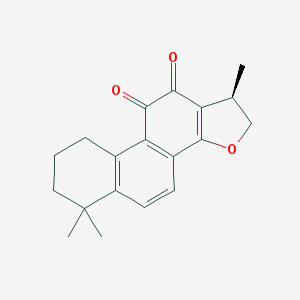 | 52.34 | 0.95 | 0.51 | 0.40 | 3.44 |
| Honghua | cytarabine | 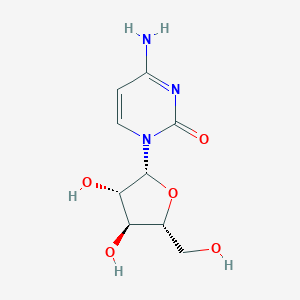 | 50.04 | 0.31 | 0.33 | 0.02 | -0.99 |
| Danshen | danshensu | 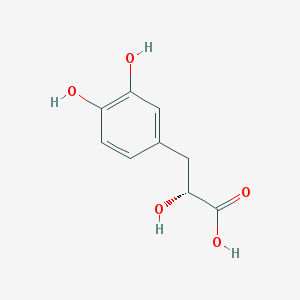 | 36.91 | -0.27 | -0.62 | 0.06 | 0.71 |
| Common | ferulic acid | 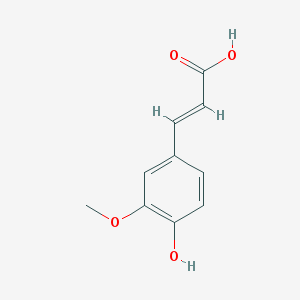 | 39.56 | 0.47 | -0.03 | 0.06 | 1.62 |
| Honghua | fluoranthene | 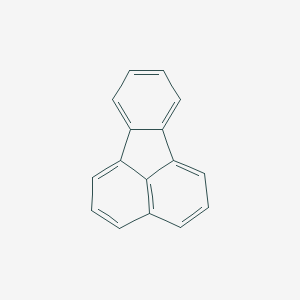 | 24.70 | 1.97 | 1.67 | 0.18 | 3.95 |
| Honghua | hydroxysafflor yellow a | 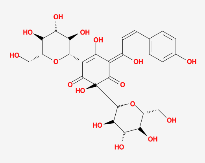 | 3.53 | -3.03 | -3.64 | 0.68 | -4.45 |
| Honghua | isoquercitrin | 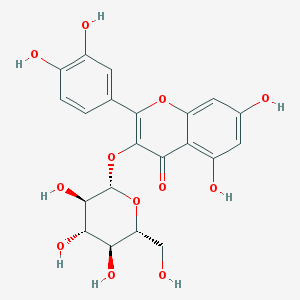 | 1.86 | -1.66 | -2.31 | 0.77 | -0.59 |
| Honghua | kaempferol | 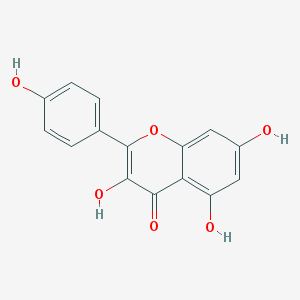 | 41.88 | 0.26 | -0.55 | 0.24 | 1.77 |
| Common | linoleic acid | 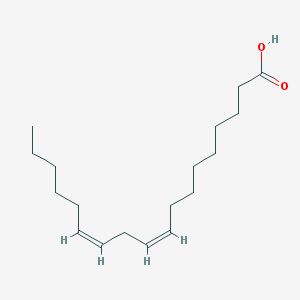 | 41.90 | 1.16 | 0.90 | 0.14 | 6.39 |
| Honghua | lutein | 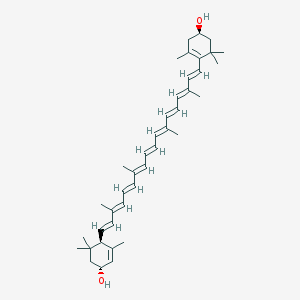 | 22.59 | 1.14 | -0.99 | 0.55 | 9.47 |
| Common | luteolin | 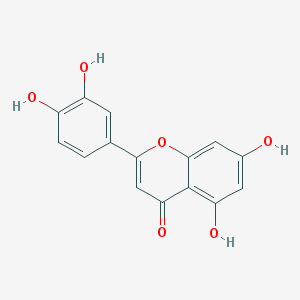 | 36.16 | 0.19 | -0.84 | 0.25 | 2.07 |
| Honghua | myricetin | 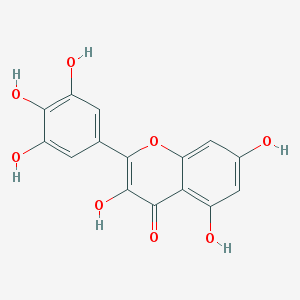 | 13.75 | -0.15 | -1.01 | 0.31 | 1.24 |
| Danshen | oleanolic acid | 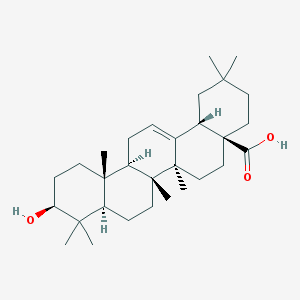 | 29.02 | 0.59 | 0.07 | 0.76 | 6.42 |
| Common | palmitic acid | 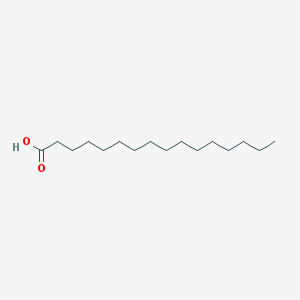 | 19.30 | 1.09 | 1.00 | 0.10 | 6.37 |
| Danshen | protocatechualdehyde | 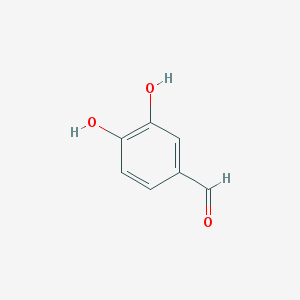 | 38.35 | 0.43 | 0.21 | 0.03 | 1.06 |
| Danshen | protocatechuic acid | 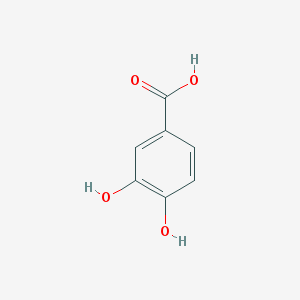 | 25.37 | 0.10 | -0.17 | 0.04 | 0.90 |
| Honghua | quercetin | 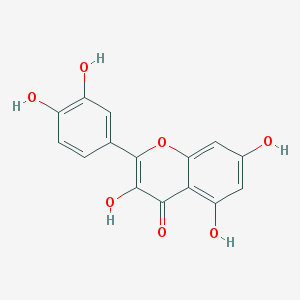 | 46.43 | 0.05 | -0.77 | 0.28 | 1.50 |
| Honghua | riboflavin | 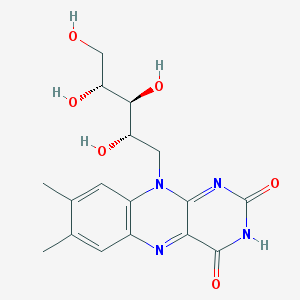 | 6.79 | -1.22 | -1.77 | 0.50 | 0.23 |
| Danshen | rosmarinic acid | 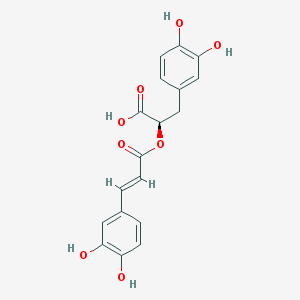 | 1.38 | -0.54 | -1.24 | 0.35 | 2.69 |
| Common | rutin | 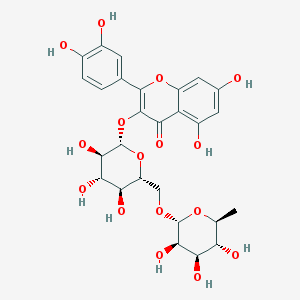 | 3.20 | -1.93 | -2.75 | 0.68 | -1.45 |
| Honghua | salicylic acid | 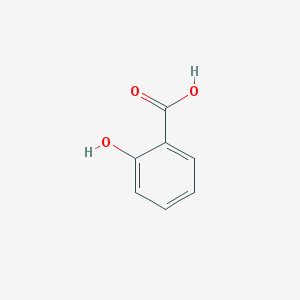 | 32.13 | 0.63 | 0.63 | 0.03 | 1.17 |
| Danshen | salvianolic acid B | 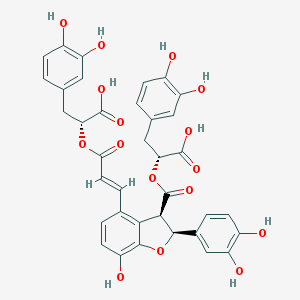 | 3.01 | -1.67 | -2.52 | 0.41 | 4.70 |
| Honghua | syringin | 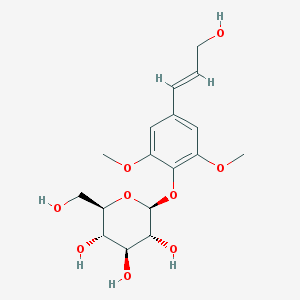 | 14.64 | -1.01 | -1.81 | 0.32 | -0.51 |
| Danshen | tanshinone IIA | 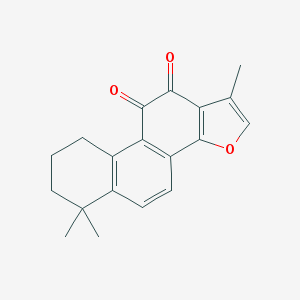 | 49.89 | 1.05 | 0.70 | 0.40 | 4.66 |
| Danshen | ursolic acid | 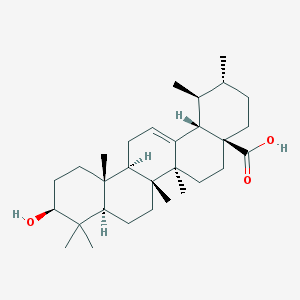 | 16.77 | 0.67 | 0.07 | 0.75 | 6.47 |
| Honghua | phytol | 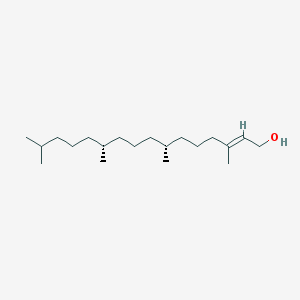 | 33.82 | 1.23 | 0.85 | 0.13 | 7.34 |

**Supplementary Table 3. Details of 37 DH ingredients and their corresponding ADME parameters predicted by TCMSP.**

1. Kramer, A., Green, J., Pollard, J., Jr. & Tugendreich, S. Causal analysis approaches in Ingenuity Pathway Analysis. *Bioinformatics (Oxford, England)* **30**, 523-530, doi:10. 1093/bio inf ormatics / btt703 (2014).

2. Amberger, J. S., Bocchini, C. A., Schiettecatte, F., Scott, A. F. & Hamosh, A. OMIM.org: Online Mendelian Inheritance in Man (OMIM(R)), an online catalog of human genes and genetic disorders. *Nucleic acids research* **43**, D789-798, doi:10.1093/nar/gku1205 (2015).

3. Liu, H. *et al.* CADgene: a comprehensive database for coronary artery disease genes. *Nucleic acids research* **39**, D991-996, doi:10.1093/nar/gkq1106 (2011).

4. Zhao, N. *et al.* Molecular network-based analysis of guizhi-shaoyao-zhimu decoction, a TCM herbal formula, for treatment of diabetic peripheral neuropathy. *Acta Pharmacol Sin* **36**, 716-723, doi:10.1038/aps.2015.15 (2015).

5. Stelzer, G. *et al.* In-silico human genomics with GeneCards. *Human genomics* **5**, 709-717 (2011).

6. Rappaport, N. *et al.* MalaCards: A Comprehensive Automatically-Mined Database of Human Diseases. *Current protocols in bioinformatics / editoral board, Andreas D. Baxevanis ... [et al.]* **47**, 1 24 21-21 24 19, doi:10.1002/0471250953.bi0124s47 (2014).

7. Ru, J. *et al.* TCMSP: a database of systems pharmacology for drug discovery from herbal medicines. *Journal of cheminformatics* **6**, 13, doi:10.1186/1758-2946-6-13 (2014).

8. Chen, C. Y. TCM Database@Taiwan: the world's largest traditional Chinese medicine database for drug screening in silico. *PloS one* **6**, e15939, doi:10.1371/journal.pone.0015939 (2011).

9. Xue, R. *et al.* TCMID: Traditional Chinese Medicine integrative database for herb molecular mechanism analysis. *Nucleic acids research* **41**, D1089-1095, doi:10.1093/nar/gks1100 (2013).

10. Chen, X. *et al.* Database of traditional Chinese medicine and its application to studies of mechanism and to prescription validation. *British journal of pharmacology* **149**, 1092-1103, doi:10.1038/sj.bjp.0706945 (2006).

11. Fang, Y. C., Huang, H. C., Chen, H. H. & Juan, H. F. TCMGeneDIT: a database for associated traditional Chinese medicine, gene and disease information using text mining. *BMC complementary and alternative medicine* **8**, 58, doi:10.1186/1472-6882-8-58 (2008).

12. Zhang, Q. Q. *et al.* Rapid separation and identification of multiple constituents in Danhong Injection by ultra-high performance liquid chromatography coupled to electrospray ionization quadrupole time-of-flight tandem mass spectrometry. *Chinese journal of natural medicines* **14**, 147-160, doi:10.1016/S1875-5364(16)60008-0 (2016).

13. Jiang, X. *et al.* Bioactivity-integrated UPLC/Q-TOF-MS of Danhong injection to identify NF-kappaB inhibitors and anti-inflammatory targets based on endothelial cell culture and network pharmacology. *Journal of ethnopharmacology* **174**, 270-276, doi:10.1016/j.jep.2015.08.026 (2015).

14. Liu, X., Wu, Z., Yang, K., Ding, H. & Wu, Y. Quantitative analysis combined with chromatographic fingerprint for comprehensive evaluation of Danhong injection using HPLC-DAD. *Journal of pharmaceutical and biomedical analysis* **76**, 70-74, doi:10.1016/j.jpba.2012.12.013 (2013).

15. Gao, L. N., Cui, Y. L., Wang, Q. S. & Wang, S. X. Amelioration of Danhong injection on the lipopolysaccharide-stimulated systemic acute inflammatory reaction via multi-target strategy. *Journal of ethnopharmacology* **149**, 772-782, doi:10.1016/j.jep.2013.07.039 (2013).

16. Zhang, Y. Y. *et al.* Effects of Danhong Injection () and its main components on anticoagulation and fibrinolysis in cultured vein endothelial cells. *Chinese journal of integrative medicine* **22**, 276-283, doi:10.1007/s11655-016-2498-x (2016).

17. Wang, D. *et al.* Vascular reactivity screen of Chinese medicine danhong injection identifies Danshensu as a NO-independent but PGI2-mediated relaxation factor. *Journal of cardiovascular pharmacology* **62**, 457-465, doi:10.1097/FJC.0b013e3182a29657 (2013).

18. Wang, X., Morris-Natschke, S. L. & Lee, K. H. New developments in the chemistry and biology of the bioactive constituents of Tanshen. *Medicinal research reviews* **27**, 133-148, doi:10.1002/med.20077 (2007).

19. Liang, W. *et al.* Quality Evaluation and Chemical Markers Screening of Salvia miltiorrhiza Bge. (Danshen) Based on HPLC Fingerprints and HPLC-MSn Coupled with Chemometrics. *Molecules* **22**, doi:10.3390/molecules22030478 (2017).

20. Zhang, L. L. *et al.* Phytochemistry and Pharmacology of Carthamus tinctorius L. *The American journal of Chinese medicine* **44**, 197-226, doi:10.1142/S0192415X16500130 (2016).
